# Living the good life? A systematic review of behavioural signs of affective state in the domestic horse (*Equus caballus*) and factors relating to quality of life. Part 2: Horse-human interactions

**DOI:** 10.1017/awf.2024.41

**Published:** 2024-10-21

**Authors:** Carol Hall, Rachel Kay

**Affiliations:** 1 School of Animal, Rural and Environmental Sciences, Nottingham Trent University, Southwell, Nottinghamshire NG25 0QF, UK; 2 National Equine Welfare Council, Slad Lane, Princes Risborough, Bucks HP27 0PP, UK

**Keywords:** animal welfare, equine ethogram, Five Domains model, human behaviour change, social licence, social needs

## Abstract

Quality of life is dependent upon the extent to which behavioural needs are met, and the balance between pleasant and unpleasant lifetime experiences. In Part II of this systematic review, articles (n = 109) relating to horse-human interactions were reviewed to identify behavioural evidence of their positive or negative impact on the horse. The number of articles (n = 22) relating to the recognition of pain in horses, indicated the importance of identifying health issues, which are also likely to increase the aversiveness of interactions. These and articles relating to emotional reactivity testing in horses (n = 19), the behaviour of horses during handling and management procedures (n = 17), behaviour of the horse when ridden (n = 17), non-procedural horse-human interactions (n = 13), horse behaviour during transportation (n = 12) and behaviour during training other than when ridden (n = 9) were reviewed. During most interactions, horse behaviour is controlled and/or restricted by the human, masking negative or positive signs, and may be confounded by factors including fear and individual differences. In situations involving freedom of movement, positive experiences of horses with humans were associated with approach behaviour, negative ones with avoidance, but training could affect both. Undoubtedly, change is needed to reduce the extent to which interactions with humans are unpleasant for the horse. Only when the needs of the horse are fulfilled and interactions with humans are predominantly pleasurable will their quality of life improve.

## Introduction

The findings presented in Part I of this review (a companion paper published simultaneously; Hall & Kay 2024) provided evidence of what horses want most (space, company, and forage) and how providing them with the opportunity to fulfil their species-specific needs is reflected in their behaviour. In agreement with Dawkins (2004), and the adaptation of the Five Domains model developed by Mellor *et al.* (2020) in the guiding principles for humane livestock farming in The Netherlands (Council on Animal Affairs 2021), it was concluded that the horse could only experience a good life if their natural behavioural needs were met. In addition, the many and varied roles of the domestic horse (*Equus caballus*) in relation to their human counterparts have resulted in multifaceted experiences, many of which could be deemed aversive to the horse. For example, transportation by road (Hall *et al.* 2020), the clipping of excessive hair (Yarnell *et al.* 2013), and the use of restrictive equipment during ridden activities (Condon *et al.* 2022). Given that an animal’s quality of life (QOL) is determined by the balance between pleasant and unpleasant experiences over time (Yeates 2016), the cumulative effect of repeated negative experiences will undoubtedly have a detrimental effect on the horse.

The importance of considering the cumulative life experiences of an animal when assessing its QOL was incorporated in the Animal Welfare Assessment Grid (AWAG), developed initially to assess the welfare of experimental animals (Honess & Wolfensohn 2010). As well as physical, behavioural/psychological, and environmental parameters, challenges associated with experimental and management procedures are included in this welfare assessment protocol (for an example of its application, see Ryan *et al.* 2021). Although the welfare parameters that form the basis of this system are not dissimilar to other approaches, the emphasis put on the impact of cumulative life experiences when assessing QOL is of relevance to all animals, including the domestic horse.

Interactions with humans occur in many different situations and could be experienced as positive or negative by the horse. However, their behavioural response may not convey this accurately, or may be misinterpreted by the human. For example, during a sham-clipping procedure, the behaviour of horses considered compliant differed from those considered non-compliant, but physiological measures (salivary cortisol) suggested that all horses found the procedure aversive (Yarnell *et al.* 2013). The interpretation of behavioural signs in the horse can be influenced by the experience and/or the role of the observer. It was found that when judging the behaviour of ridden horses for signs indicative of positive or negative subjective state, results varied between different equestrian professions (Hall *et al.* 2014).

When assessing the impact of human interactions on the QOL of the horse, it is important to consider the first of the questions posed by Dawkins (2004): is the animal healthy? Although physical health can, to a certain extent, be measured, behavioural signs of underlying pain may be missed. To ensure that subsequent interactions are not associated with unpleasant painful experiences, resulting in them becoming aversive to the horse, further evidence to facilitate the recognition of behavioural signs of pain is required.

As reported in Part I (Hall & Kay 2024), a systematic review of scientific evidence was conducted to derive observable, evidence-based behavioural measures of positive or negative affective state and factors that relate to QOL in the domestic horse. In Part II, the retained articles relating to horse-human interactions will be reviewed to identify behavioural evidence of the positive or negative impact of these on the horse. As used in this review, the term horse-human interaction refers to the many and varied situations where the behaviour of the horse is determined by human action, and where accurate interpretation of horse behaviour should guide subsequent interactions. Features of these interactions, associated with whether they are experienced as positive or negative, will be identified to facilitate the development of less-aversive approaches. The aim of this review is to promote more positive horse-human interactions that will contribute to a better life for horses. In combination with the findings of Part I of this systematic review (Hall & Kay 2024), the changes in management and training that are required for horses to live a good life will be explored.

## Materials and methods

During February 2023, a systematic literature search of five electronic databases (Science Direct, PubMed, Scopus, Web of Science and Pubpsych) was performed. Two separate search queries were specified to ensure the potential inclusion of behaviour indicative of both negative and positive affect:

Search 1: ‘(emotion* OR affect* OR cognit*) AND (behav* OR welfare) AND (horse OR pony OR equine)’

Search 2: ‘(stress AND behav*) AND (horse OR pony OR equine)’

Following a preliminary assessment of the relevance of each peer-reviewed article, those reporting primary research findings with a clear account of the methods used (experimental and/or observational), a subject number greater than four, and justification for any subsequent attribution of affective valence to aspects of recorded behaviour, were retained. No criteria relating to the date of publication were used in the searches. Only articles involving horses and/or ponies were retained.

The following information was extracted from the retained articles: the scenario involved, the behaviour recorded, the subjective experience attributed to specific behaviours together with the supporting evidence for this, and factors that affected the behaviour expressed. Features of the study design, including details of the horses/ponies involved in each study, behaviour recorded, and the nature of the supporting evidence are available in the Supplementary material.

See Part I (Hall & Kay 2024) of this systematic review for full details of the methods used to select the articles for retention, and the data extracted from these. Articles relating to the fulfilment of species-specific needs (n = 70) were included in Part I, and those relating to horse-human interactions (including all management and training procedures) (n = 109) are included here in Part II.

## Results and Discussion

The search terms used in this systematic review identified articles relating to most aspects of the life of the domestic horse. The study scenarios involving horse-human interactions (as defined above) and the number of articles per scenario are shown in Table 1. The scenarios are listed in descending order according to the number of articles retained. The number of scenarios identified during this literature search exemplifies the multi-faceted life of the domestic horse and the demands associated with their role as a sporting, leisure, or companion animal. The many aspects of management and training included in the scenarios listed in Table 1 reflect both the multiple ways in which domestic horse welfare may be compromised, but also the potential for reviewing all aspects of horse husbandry to promote a better quality of life. The behaviour attributed to affective state and the factors affecting this behaviour are reported for each separate scenario. Further details of the experimental design used in the articles retained under each scenario are available in the Supplementary material (Tables S2.1–S2.7). General behavioural signs of affective state during interactions with humans and the subsequent welfare implications are discussed below.

**Table 1.** Study scenarios relating to horse behaviour during horse-human interactions identified in the literature search, the related table in the Supplementary material and the number of articles retained per scenario. The scenarios are listed in descending order according to the number of articles retained

Type of study scenario	Table number in Supplementary material	Number of articles retained
Behavioural signs of pain	S2.1	22
Tests of emotional reactivity	S2.2	19
Handling and management procedures	S2.3	17
Behaviour of the horse when ridden	S2.4	17
Horse–human interactions (non–procedural)	S2.5	13
Transport	S2.6	12
Training other than when ridden (methods and equipment)	S2.7	9

### Behavioural signs of pain in the horse

The recognition of behavioural signs of pain in the horse is key to answering the first of the questions posed by Dawkins (2004): is the animal healthy? The importance of identifying signs that the horse is experiencing or anticipating pain was reflected in the number of articles on this topic that resulted from the search terms. A total of twenty-two articles relating to behavioural signs of pain associated with either specific health issues or in general were retained. Pain was induced in five of the studies, with the aim of identifying behavioural signs to facilitate the recognition of pain for future application. Behaviour recorded in four of these studies (Bussières *et al.* 2008; Grint *et al.* 2017; Reid *et al.* 2017; Egan *et al.* 2021) is shown in Table 2. In the study by Carvalho *et al.* (2022) induced, low-grade, inflammatory pain was not detectable via analysis of facial expression (and consequently is not included in Table 2). Low-level pain was not found to affect typical feeding behaviour, but behavioural variability increased as horses recovered from induced bilateral lameness resulting from mild inflammation in the joints, although this varied for individual horses (Egan *et al.* 2021). There was also variation in individual behavioural responses to the induced pain and recovery, with no generic ‘normal’ behaviour identified (Egan *et al.* 2021).

**Table 2.** Behavioural signs of pain in the horse (in relation to specific health issues and in general)

Health issue (source of pain)	Behaviour associated with health issue	References
Induced pain:		
Orthopaedic	Avoidance of tactile pressure, pawing on floor, kicking at abdomen, head movements.	Bussières *et al.* 2008
Induced bilateral synovitis of intercarpal joints	Behavioural variability increased as horses recovered from the low–level pain, but there was individual variation.	Egan *et al.* 2021
Pressure testing	Flattening ears back, turning head, avoidance foot lifting, biting at self.	Grint *et al.* 2017
Neck pinch	Reduced locomotion, reduced contact seeking behaviour.	Reid *et al.* 2017
Abdominal pain:		
Low forage diet (resulting in potential pain)	Increased vigilance in novel situations and in the company of an unfamiliar horse.	Destrez *et al.* 2015
Gastric ulcers	Increased speed of eating, pawing at floor.	Malmkvist *et al.* 2012
	More frequent eating bouts, abdominal kicking, tail swishing, tongue activity, pawing.	Perron *et al.* 2023
Pressure in gastrointestinal tract	Looking at flank, pawing, restlessness, stretching, vocalising, getting up and down, rolling.	Pritchett *et al.* 2003
Post–operative pain:		
General	Reduced time spent interacting with a novel object and reduced response to auditory startle test one day post–surgery/anaesthesia.	Dodds *et al.* 2017
Post castration	Stiffly backwards ears, orbital tightening, tension above the eye area, prominent strained chewing muscles.	Dalla Costa *et al.* 2018
	Reduced time spent eating/drinking, looking round at wound, retract pelvic limb, explore penis, looking to back of stable.	Trindade *et al.* 2021
Emergency colic surgery	No movement/reduced locomotion post–surgery.	Pritchett *et al.* 2003
Chronic back pain:		
Severe vertebral problems	Increased aggression towards humans.	Fureix *et al.* 2010
Lesser vertebral problems	Reduction in positive responses to/interactions with humans.	Fureix *et al.* 2010
Chronic back disorders	Neck posture (concavity of neck top line) associated with vertebral disorders.	Lesimple *et al.* 2012
Back/vertebral disorders	Level of disorder / pain positively correlated with lack of attention/engagement.	Rochais *et al.* 2016a
Osteoarthritis:		
Severe osteoarthritis	Reduced time spent in recumbency.	Oliveira *et al.* 2022
Mild osteoarthritis	Increased time spent in recumbency.	Oliveira *et al.* 2022
Laminitis:	Weight shifting between contralateral limbs.	Rietmann *et al.* 2004a
Oral pain (dental/cheek teeth):	Eating: eats hay slowly, pauses while eating, drops hay, dips hay in water.	Laukkanen *et al.* 2023
	Ridden (bit): opens mouth, evades the bit, tongue lolling, head shaking, uneven left/right rein contact.	Laukkanen *et al.* 2023
	General behaviour: head shaking, halitosis, lack of interest in surroundings.	Laukkanen *et al.* 2023
Ocular pain:	Head below withers, ears back, lacrimation (tear production), blepharospasm (abnormal contraction of the orbicularis oculi muscle), avoiding physical contact.	Ortolani *et al.* 2021
General pain:		
From poor physical health, sickness, exhaustion, chronic pain	Depression like symptoms, lack of alertness, avoidance of human contact.	Burn *et al.* 2010

The differentiation between behaviour resulting from anxiety or pain was explored by Reid *et al.* (2017), by testing responses to anxiety-provoking situations (social isolation) and/or a pain-eliciting stimulus (neck pinch). Pain alone resulted in reduced locomotion and reduced contact-seeking behaviour, whereas anxiety resulted in increased locomotion, increased vocalisation, restlessness, and contact-seeking behaviour. Anxiety and pain together caused restlessness. The authors concluded that social isolation (and potentially other sources of anxiety) may affect behaviour associated with pain responses, or potentially be the cause of behaviour typically cited as being indicative of pain (Reid *et al.* 2017).

Behavioural signs of pain were also found to be confounded by associated stressors by Erber *et al.* (2012). A comparison between the effect of hot branding and microchipping (as a means of identification) on the behavioural and physiological responses of foals concluded that both processes were stressful and that the associated restraint was a major stressor. No specific behavioural details were provided but the authors noted that painful procedures should always be avoided, particularly in young animals where early experiences may have an impact both on future pain perception, and on responses to handling and other management procedures (Erber *et al.* 2012). Dalla Costa *et al.* (2018) found that certain facial expressions included in the Horse Grimace Scale (developed as a means of identifying pain in horses) were also associated with fear-eliciting situations (stiffly backward ears and prominent strained chewing muscles). Given that facial characteristics vary according to breed and type of horse (Hintze *et al.* 2016) and Carvalho *et al.* (2022) found that low-grade pain was not identifiable from facial expression, interpretation of these subtle behavioural signs should be treated with caution.

Evidence suggests that anticipation of pain and pain in general results in immobility and the avoidance of human contact (Pritchett *et al.* 2003; Burn *et al.* 2010; Fureix *et al.* 2010; Reid *et al.* 2017). Severe and/or acute pain was recognised by the horses’ behavioural attempts at avoiding contact with humans (Burn *et al.* 2010; Fureix *et al.* 2010), lack of any movement likely to worsen the pain (Pritchett *et al.* 2003; Reid *et al.* 2017) and decreased time spent eating (Trindade *et al.* 2021). Monitoring any changes in these features of behaviour can provide important insights into the ongoing health status of the horse.

Certain behaviours have been shown to be associated with specific health issues (as shown in Table 2), but individual variation occurs. For example, although Oliveira *et al.* (2022) found that the severity of osteoarthritis affected the time horses spent in recumbency during hospitalisation (see Table 2), Kelemen *et al.* (2021) noted that neither age nor lameness due to chronic orthopaedic disease had an impact on the time horses spent recumbent.

Behavioural signs of pain in the horse (in relation to specific health issues and in general) are shown in Table 2. See Table S2.1 in the Supplementary material for details of each study.

#### In summary

The recognition of behavioural signs of pain in horses remains one of the most important areas of research in terms of improving their quality of life. Distinguishing between behaviour indicative of pain and that associated with confounding factors including fear and anxiety, isolation, anticipation of pain and other treatment-related stressors, is further complicated by individual variation. Familiarity with an individual horse may be valuable in determining whether changes in behaviour signify underlying pain. In addition, where specific behavioural issues occur, for example, avoidance behaviour during preparation for ridden work, these may relate to anticipation of pain or discomfort and should not be ignored. See *Behaviour of the horse when ridden.*

### Tests of emotional reactivity in the horse

Challenges to human safety during interactions with horses, both during handling and ridden work, have been associated with sudden/unexpected, often intense, behavioural responses of the horse (Górecka-Bruzda *et al.* 2015; Lansade *et al.* 2019). Tests of emotional reactivity to various acute stressors (for example, social separation/isolation, startling/sudden occurrences, and novelty) have been developed to explore factors affecting such behavioural responses, with the aim of enabling better assessment and selection of horses and reducing human risk. Nineteen articles reporting the results of experimental tests of emotional reactivity, including novel object, novel environment, startle, and isolation tests, were retained. Factors found to affect reactivity were age (Baragli *et al.* 2014; Bulens *et al.* 2015; Lee *et al.* 2021), breed/type (Lee *et al.* 2021), type of work/role (Hausberger *et al.* 2011; Mendonça *et al.* 2019), the effect of company (Lansade *et al.* 2012; Rørvang & Christensen 2018; Ricci-Bonot *et al.* 2021), training (Squibb *et al.* 2018) and laterality (Larose *et al.* 2006; De Boyer Des Roches *et al.* 2008; Baragli *et al.* 2021). As above, individual differences in the behavioural reactions elicited by these tests were reported by Lansade *et al.* (2008), Pérez Manrique *et al.* (2019) (see also *Behaviour associated with weaning* in Part 1; Hall & Kay 2024), Safryghin *et al.* (2019), and Manrique *et al.* (2021).

#### Fear and frustration

The effect of experimentally induced negative or positive situations on learning, using startle tests and food, respectively, was reported by Fortin *et al.* (2018) and Olczak *et al.* (2021). Improved cognitive flexibility in an instrumental learning task was associated with an environment where positive events had occurred (Fortin *et al.* 2018). Fear generated by waving a plastic bag was associated with an increase in the number of trials required to reach criterion in a clicker-training (positively reinforced) task (Olczak *et al.* 2021). The inability to succeed at a task, regardless of training, has been shown to produce behavioural signs of frustration and an increase in HR (Rørvang *et al.* 2021). These findings have important implications for horse training in general, where potential sources of fear should be avoided, and the tasks involved must be within the capabilities of the horse.

#### Age, training, and role of horse

Age-related differences in both behavioural and physiological responses in a visual startle test were found by Baragli *et al.* (2014). Young horses displayed a greater behavioural response (avoidance) and more exploratory behaviour than older horses, but older horses had a more pronounced physiological response (decrease in HRV). Similarly, during novel object tests, younger horses showed a more pronounced behavioural reaction than older horses (Bulens *et al.* 2015), whereas older horses showed more physiological signs of anxiety (decrease in HRV; Lee *et al.* 2021).

Inconsistency between physiological (HR, HRV, rectal temperature, eye temperature) and behavioural signs of anxiety in adult horses during novel handling procedures (walking over a tarpaulin and through plastic streamers) was attributed to the overriding effect of training on the behavioural response by Squibb *et al.* (2018), which may at least in part explain the age-related differences. These findings suggest that because adult horses do not always behave in a way that reflects their emotional response, the potentially negative impact of events may be underestimated. In part, this reduced behavioural response could be explained by differing levels of training, as suggested by Squibb *et al.* (2018). Early restrictive training of neonates was indeed shown to subsequently reduce active locomotor responses to social separation and novelty (Durier *et al.* 2012).

The extent to which training is responsible for individual differences in behavioural responses is unclear. There is some evidence of consistent individual differences in responses to social separation (Pérez Manrique *et al.* 2019; Manrique *et al.* 2021) and the trait of fearfulness (Lansade *et al.* 2008). Regardless of whether behavioural tendencies are innate or acquired, individual differences in the extent to which observed behaviour reflects underlying affective state must be recognised to reduce the risk of misinterpreting the impact of events and/or procedures on the horse.

The type of work (role) the horse is involved in has been associated with behavioural differences in responses to novel object, startle, isolation, and novel environment tests (Hausberger *et al.* 2011). In agreement with Brubaker *et al.* (2021) and Lerch *et al.* (2021) (see Table 5[b]), Mendonça *et al.* (2019) found that horses involved in Equine Assisted Service work showed behavioural signs of apprehensiveness (and physiological signs including decreases in HRV) in relation to human contact during a novel test. In comparison, dressage horses showed more exploratory behaviour and both dressage and eventing horses were quicker to approach a human during testing. Showjumping horses responded less to novelty than those in other working groups (Mendonça *et al.* 2019). Breed/type of horse (which is also likely to vary according to the role of the horse) may also be a contributory factor. For example, Lee *et al.* (2021) found that the physiological response was most strongly correlated with behaviour in thoroughbred mares compared to other breeds.

#### Consistency of behavioural responses

Individual differences in behavioural responses have, in some cases, been found to be consistent characteristics (temperament, personality). Foal responses to separation from the mare (from birth to six months) were found to be consistent with responses to group separation at nine to twelve months (including frequencies of vocalisation, head tossing, high head carriage and locomotion) (Pérez Manrique *et al.* 2019; Manrique *et al.* 2021). Although consistent, repeatable, fear-related behavioural responses to novel object and startle tests were recorded (from eight months to two and a half years of age) by Lansade *et al.* (2008), there was no correlation between HR and behaviour. The authors concluded, however, that the consistency in behaviourally measured fear responses indicated that the ‘fearful’ trait varies with individuals and is present from an early age (Lansade *et al.* 2008). However, in adult horses, Safryghin *et al.* (2019) did not find repeatable behavioural responses to different tests (novel object and pre-feeding test) and, again, physiological responses (HR) were not consistent with observed behaviour. It is likely that both innate and acquired individual differences affect reactivity.

#### Company

The presence of other horses has been shown to affect behavioural responses in different ways according to context. Lansade *et al.* (2012) found that social isolation for a period of eleven days resulted in reduced reactivity to a novel object in yearling foals and improved learning in a motor task (moving forwards and backwards). A possible explanation for the decreased reactivity is that the absence of a companion resulted in a reduction in the extent to which emotion was expressed and a lack of opportunity for anxiety to be transferred between individuals. Also, a period of isolation would result in an absence of subsequent separation anxiety distracting the foal from learning a task or being an additional factor in response to a novel object. Adult horses with signs of withdrawal/depression were found to be less reactive to auditory stimuli than non-withdrawn horses (Rochais *et al.* 2016b), which may also be a contributory factor in the reduced reactivity of isolated foals. An example of how reduced emotional responses can also be transferred was reported by Rørvang and Christensen (2018) who found a reduction in behavioural and physiological signs of fear in response to a startle test when naïve horses were in the presence of a habituated demonstrator. However, Ricci-Bonot *et al.* (2021) did not find that the presence of a companion affected the behavioural response of horses in a startle test, but this did result in a quicker physiological recovery (HR return to baseline). The reverse occurred in a novel object test (no effect on HR but a reduction in behavioural reactivity), and the authors suggested that this reflects the increased effect of social buffering in situations where greater contextual processing is facilitated (Ricci-Bonot *et al.* 2021).

#### Laterality

Asymmetry of brain function and behaviour in vertebrate animals, including the horse, allows for specialisation and has also been found to be associated with emotional responses. In general, the right hemisphere is involved in rapid, emotional responses, and the left more in decision-making (Rogers 2002). The resultant side-related differences in behaviour, or lateralisation, and the potential association between lateral preferences and affective valence (for example, see Ahern & Schwartz 1979) was investigated but no consistent patterns emerged. De Boyer Des Roches *et al.* (2008) found a slight tendency for horses to explore what was deemed a negative stimulus (shirt associated with veterinary surgeon) with their left eye. Breed differences in preferred eye for inspection of a novel object were found by Larose *et al.* (2006), with French Saddlebreds using their right eye more and Trotters their left. Baragli *et al.* (2021) found individual differences in which eye was preferred when inspecting a novel object, but no consistent group-level differences were found. There have been some findings that suggest an association between affective state and lateral preferences (see Marr *et al.* 2018; Marliani *et al.* 2022; Hall & Kay 2024), but the latter is also likely to be influenced by human handling and training (see Table 5[b] and Sankey *et al.* 2011). Although no consistent pattern of lateralisation was found, lateral differences in response to handling, training, and management procedures should be considered at an individual horse level. See Table S2.2 in the Supplementary material for details of the articles retained relating to this scenario.

#### In summary

Although factors such as age, breed/type and training were associated with the extent to which behavioural reactions occurred in response to various sudden or acute stressors, the potential for such behaviour remains in all horses. However, the results of the retained studies suggest some ways in which both the risk to human safety and negative impact on the horse can be reduced. Gradual habituation to potentially negative experiences, such as separation and isolation, careful introduction to potentially challenging situations, and the company of a more experienced, habituated animal should all lessen the negative impact on the horse. In terms of human safety, the careful pairing of less-reactive horses with inexperienced humans will be mutually beneficial. Also, an increased awareness of what constitutes an acute stressor for the horse and likely behavioural responses would be of value to inexperienced and potentially more experienced humans alike.

### Behaviour of the horse during handling and management procedures

There were seventeen articles retained that had a focus on handling and management procedures. The predominant behavioural measures used were the extent to which the horse tried to avoid the procedure or was relaxed/approached the human carrying out the procedure, and/or whether the horse showed behavioural signs indicative of stress. Approach/avoidance behaviours were concluded as signifying positive or negative responses, respectively. Behavioural signs of stress were identified based on past studies (for example, Young *et al.* 2012) and were believed to indicate negative affective state. Behaviour indicative of positive or negative responses to handling and management procedures are shown in Table 3(a). Included in Table 3 are behaviours during handling tests that were associated with different home environments (Harewood & McGowan 2005; Yarnell *et al.* 2015) See Part I of this review, *The home environment* (Hall & Kay 2024).

**Table 3.** (a) Behaviour during handling and management procedures indicative of affective state (positive or negative), supporting evidence*, and b) factors affecting this behaviour

3(a) BEHAVIOUR DURING HANDLING AND MANAGEMENT PROCEDURES				
	Signs of positive affective state	Basis of justification	Signs of negative affective state	Basis of justification
Behavioural states				
Feeding	Increased time spent feeding	PS, PHYS (Guinnefollau *et al.* 2021)	Reduced time spent feeding	PS, PHYS (Guinnefollau *et al.* 2021)
Behavioural events				
Facial expression	Decreased angle between the highest point of the wrinkle above the eye and the horizontal plane of the eyeball	+ve SIT (Hintze *et al.* 2016)	Increased angle between the highest point of the wrinkle above the eye and the horizontal plane of the eyeball	–ve SIT (Hintze *et al.* 2016)
			Prominent strained chewing muscles	PS, –ve SIT (Dalla Costa *et al.* 2017)
			Decreased number of full and half eye blinks; increased number of eyelid twitches	PS, –ve SIT (Lelláková *et al.* 2021)
Head/neck movements	Higher frequencies of the neck held at a medium height, half closed eyes, lips extended forwards/twitching and upper lip extended forwards and immobile. Mutual grooming Lip movements Head lowering, licking, chewing, yawning	+ve SIT, +ve SOC (Lansade *et al.* 2018) +ve SOC (Lansade *et al.* 2018; Lansade *et al.* 2019); PHYS (McBride *et al.* 2004) PHYS, +ve SOC (Birt *et al.* 2015)	Higher frequencies of the neck held high, the eyes wide open, the whites of the eyes visible, the lips held straight or contracted, and ears held asymmetrically. Biting/threatening to bite handler Elevated head and lateral movement away from handler Head tossing, shaking, nodding, turning Head shaking Stiffly backwards ears Lip licking/chewing	PS, –ve SOC (Lansade *et al.* 2018; Lansade *et al.* 2019) PS, AVO, –ve SOC (Harewood & McGowan 2005) PS, PHYS, –ve SOC (Yarnell *et al.* 2015) –ve SIT (Ali *et al.* 2017; Górecka–Bruzda *et al.* 2017) PS, –ve SOC (Whitaker *et al.* 2009; Dalla Costa *et al.* 2017) PHYS, –ve SOC (Guinnefollau *et al.* 2021) –ve SIT, –ve SOC (Watson & McDonnell 2018)
Body movements	Approaching human handler, relaxed behaviour Standing still, relaxed Lateral movement of hindquarters	APP, +ve SOC (Lansade *et al.* 2018; Lansade *et al.* 2019) +ve SOC (Harewood & McGowan 2005) PHYS (McBride *et al*. 2004)	Withdrawal from handler; Whole body movements/jerks Pulling away from handler Increased frequency of body movements Whole body freezing Avoidance behaviour Moving away from handler	AVO, –ve SOC (Ali *et al.* 2017; Górecka–Bruzda *et al.* 2017) AVO, –ve SOC (Watson & McDonnell 2018) –ve SOC, AVO (Lansade *et al.* 2018) PHYS, AVO, –ve SOC (Yarnell *et al.* 2015)
Leg / hoof movements			Rearing Stepping away Kicking Kicking at handler Stomping, kicking, pawing Pawing	–ve SOC, AVO (Górecka–Bruzda *et al.* 2017) –ve SOC(Lansade *et al.* 2018); –ve SOC, AVO (Harewood & McGowan 2005) AVO, –ve SIT (Padalino *et al.* 2019)
Tail movements			Swishing tail	–ve SOC (Watson & McDonnell 2018); –ve SIT (Padalino *et al.* 2019; Guinnefollau *et al.* 2021)
Vocalisations			Snorting and other vocalisations Increased vocalisation	–ve SOC (Watson & McDonnell 2018) –ve SIT, –ve SOC (Harewood & McGowan 2005); –ve SIT, AVO (Guinnefollau *et al.* 2021)
Defaecation			Occurrence of defaecation during handling procedure	–ve SIT (Watson & McDonnell 2018)

3(b) FACTORS AFFECTING BEHAVIOUR		
Home environment	Horses housed singly/in social isolation were found to display more avoidance behaviour during handling than those housed with access to social contact.	(Yarnell *et al.* 2015)
Type of procedure	Procedures involving physical manipulation (for example, hoof trimming, invasive veterinary procedures) were found to be more aversive for young, inexperienced horses than those that did not (for example, conformation measurements).	(Górecka–Bruzda *et al.* 2017)
Duration of procedure	In naïve, pre–weaned horses, the duration of the procedure was an important factor in determining the severity of the stress response (positive association duration: severity)	(Górecka–Bruzda *et al.* 2017)
Human behaviour	In naïve, pre–weaned horses, when procedures that resulted in avoidance behaviour were carried out by experienced handlers, no long–term negative effects in relation to procedural sensitisation or avoidance of human contact were found. During common procedures such as grooming, humans were found to disregard behavioural signs of comfort/discomfort in the horse. If grooming practice takes account of the preferred body areas and indications that the horse is responding in a positive or negative way, contact–seeking rather than avoidance behaviour was facilitated. Adopting a responsive rather than fixed approach to management procedures was found to promote positive experiences for both horse and human.	(Górecka–Bruzda *et al.* 2017) (Lansade *et al.* 2018; 2019)
Familiarity with human handler	Horses handled by one familiar human were less resistant to approaching/being touched by a novel object, and to walking on a novel surface than horses handled by multiple handlers. The longer the horse had been handled by one familiar human, the less resistant they were. Familiarity with handler may mask behavioural signs of fear.	(Liehrmann *et al.* 2022) (Marsbøll and Christensen 2015)
Physical restraint	Physical restraint (commonly applied during management procedures) causes a stress response prior to the procedure. The use of non–confrontational handling techniques (for example, the use of food or positive tactile stimulation to distract the horse) in mildly aversive procedural situations can lessen the need for increased physical restraint and consequently reduce the negativity of the experience for the horse. Negative responses to procedures are masked by physical restraint and more subtle behavioural signs (such as facial expression, eyelid twitches and eye blinks) should be used to monitor reactions.	(Guinnefollau *et al.* 2021) (Watson & McDonnell 2018) (Lansade *et al.* 2018; Lelláková *et al.* 2021)
Preparatory training	Slow introduction to procedures/methodological training/habituation reduces the negative impact of management procedures on the horse and associated behaviour. Prolonged handling of foals (forced immobility and extensive rubbing/physical contact) immediately after birth was found to result in reduced locomotory responses to social separation and novelty after 1–2 years.	(Guinnefollau *et al.* 2021) (Durier *et al.* 2012)

* Key to supporting evidence: Past studies (PS), physiological measures (PHYS), assumption of associated pain (PIP), situations deemed positive or negative (+ve/-ve SIT), positive or negative social interactions (horse/human) (+ve/-ve SOC), approach/avoidance (APP/AVO), choice (PREF).

#### Handling and management procedures

The handling/management procedures included ear clipping with or without the application of a lip twitch (Ali *et al.* 2017), grooming (Hintze *et al.* 2016; Lansade *et al.* 2018, 2019), blood and saliva sampling (Lelláková *et al.* 2021), hoof trimming, blood sampling and microchip implantation in pre-weaned foals (Górecka-Bruzda *et al.* 2017), early intensive handling of neonates (Durier *et al.* 2012), massage (McBride *et al.* 2004) and the application of Flowtrition soft touch therapy (Birt *et al.* 2015), participation in practical training sessions for veterinary students (horse handling and mare reproductive/medical rectal examination) (Guinnefollau *et al.* 2021), and anthelmintic administration (Whitaker *et al.* 2011).

Behaviours classed as approach/positive included movement of lips, attempting to groom human, leaning towards/against human (McBride *et al*. 2004), and attempting to contact, nibble handler (Lansade *et al.* 2018). Avoidance/negative behaviours included moving away, threatening, biting human (Lansade *et al.* 2018), withdrawal, head shaking, rearing (Górecka-Bruzda *et al.* 2017) and head and whole-body movements away from the handler (Ali *et al.* 2017). Behavioural signs of approach/relaxation during massage at preferred sites (McBride *et al*. 2004) and the application of soft-touch therapy (Birt *et al.* 2015) were associated with reduced heart rate. Increased HR was associated with some potentially aversive procedures that resulted in avoidance behaviour, including hoof trimming in foals (also associated with increases in salivary cortisol: Górecka-Bruzda *et al.* 2017), and ear clipping without the use of a lip twitch (Ali *et al.* 2017). Ear clipping was also associated with lower HRV when no lip twitch was applied than when carried out with one (Ali *et al.* 2017).

Behavioural responses to some intrinsically aversive procedures may be reduced by environmental constraints. For example, an increase in heart rate was recorded in horses restrained in stocks for veterinary student training in rectal examination, but only minimal negative behavioural signs such as backward weight shifts, ears back and reduced time spent eating hay were recorded (Guinnefollau *et al.* 2021). The lack of overt behavioural signs may not represent underlying affective state but result from repeated restraint.

#### Familiarity and behaviour of human handler

The impact of the behaviour and familiarity of the human handler on the horse’s response to procedures and novel or fear-eliciting situations was the focus of four retained articles. In the study by Watson and McDonnell (2018) the application of handling interventions (eye rubbing, wither scratching and feed presentation) was shown to reduce avoidance behaviour during a simulated healthcare scenario, although no reduction in heart rate compared with the ‘no intervention’ trials was found. Food presentation was the most effective intervention in terms of reducing the frequency of avoidance/stress responses (Watson & McDonnell 2018). Although grooming technique (gentle responsive grooming compared with fixed pattern grooming) resulted in behavioural differences, with the gentle technique associated with contact-seeking behaviour, no effect on physiological measures was found (HR, HRV, plasma cortisol) by Lansade *et al.* (2018). However, eleven sessions of gentle grooming reduced oxytocin levels (Lansade *et al.* 2018). In potentially fear-eliciting situations (for example, fear tests involving the negotiation of ground-level novel objects), avoidance behaviours were reduced when the horse was with a familiar (as opposed to unfamiliar) handler, although this had no effect on the associated heart rate (Marsbøll & Christensen 2015).

Although some association between behaviour and physiological responses was found, this was not consistent across studies. The length of time horses had been with a handler was shown to relate to their behaviour in tests involving novel objects and novel surfaces. The longer the horse-human relationship, the less avoidance behaviour (Liehrmann *et al.* 2022). However, no physiological measures were included in this study, so whether the familiarity with the handler reduced the potential fear/response to novelty, or just how this was expressed behaviourally is unclear. However, there is the potential for familiar handlers to communicate more effectively with the horse and that familiarity may also facilitate a reduction in anxiety.

Factors affecting behaviour during handling and procedures are shown in Table 3(b).

#### Horse facial expression in response to handling

Facial expression in response to handling was assessed in three retained articles. Grooming was used as a potentially positive experience for the horse by Dalla Costa *et al.* (2017), to test the application of the horse grimace scale (HGS) to identify affective state. The only facial expression found to be associated with affect was tension in the chewing muscles and changes in ear posture in negative/fear-eliciting situations (Dalla Costa *et al.* 2017). Grooming may not be a wholly positive experience for the horse (see also Young *et al.* 2012, where salivary cortisol levels in response to grooming were not significantly lower than when exposed to the noise of fireworks and were higher than those relating to clipping and social isolation), and it is often associated with preparation for ridden work (which may be a negative experience). Further investigation of the potential for facial expression to identify affective state may be warranted. Hintze *et al.* (2016) found consistent differences in eye wrinkle expression in relation to procedures deemed to be associated with affective state (positive: grooming, food anticipation; negative: food competition, waving a plastic bag). However, variation in relation to breed or type was found by Schanz *et al.* (2019), and again, assumptions were made regarding the valence of the subjective experience of the horse, so further validation is required.

Sources cited for the ethograms used varied according to the focus of the study and type of behaviour recorded. The most prevalent citation for behaviour indicative of stress (n = 3 studies) was Young *et al.* (2012), including by Padalino *et al.* (2019) to record stress-related behaviour when horses were tied up in full sun (average temperature of 31.7°C on day 1, 26.0°C on day 2) for two hours. Unsurprisingly, all horses displayed signs of discomfort (pawing, tail swishing, repetitive head movements, licking and chewing, and biting at flies) and increases in heart rate over and above normal values for horses (Padalino *et al.* 2019). The aim of this study was to test the effect of cotton rugs to reduce the negative impact of hot, sunny weather. A better approach in terms of minimising the distress caused to the horses in this study would have been to provide a shade option and compare the use of this between horses with or without rugs. The authors do conclude that shade should be provided for horses at ambient temperatures of greater than 25°C.

Study details for the articles relating to this scenario are provided in Table S2.3 in the Supplementary material.

#### In summary

The overt behavioural responses to handling and procedures have been shown to be affected by training and movement restraint. Where there were opportunities to either approach or avoid the procedure and associated human handler, these could be assumed as indicating the horse had a positive or negative expectation of the outcome, respectively. However, when procedures are associated with attempted avoidance, and likely to be aversive in some way, methods of restraint and/or extensive training are often used with the result that behavioural responses are reduced. Inconsistencies with physiological measures have indicated that such procedures remain aversive, although this is not reflected in the behaviour of the horse. An evaluation of how these procedures could be adapted to at least make them less unpleasant for the horse (and ideally more pleasant) is now required.

### Behaviour of the horse when ridden

A total of seventeen articles with a focus on ridden behaviour were retained. These included studies assessing the effect of head/neck position on behaviour when ridden (von Borstel *et al.* 2009; Christensen *et al.* 2014), comparative approaches to training (Visser *et al.* 2009; Mendonça *et al.* 2020), potential stress associated with therapeutic ridden sessions (Kaiser *et al.* 2006; McDuffee *et al.* 2022), the effect of different riders (Christensen *et al.* 2021) and behavioural indicators of pain (Dyson & Pollard 2021) or anticipated pain (Dyson *et al.* 2022) associated with ridden work (see also *Behavioural signs of pain in the horse*). In most studies, the behaviour being assessed was that which relates to how the horse responds to the rider’s signals. The focus was on behaviour that deviated from that desired by the rider, being referred to as indicative of stress, discomfort, fear, frustration, and conflict (Kaiser *et al.* 2006; Visser *et al.* 2009; von Borstel *et al.* 2009; Christensen *et al.* 2014; Jastrzębska *et al.* 2017; Ruet *et al.* 2020; Christensen *et al.* 2021; McDuffee *et al.* 2022). Consequently, the behaviours indicative of negative affect when the horse is ridden as shown in Table 4(a) far outweigh behavioural signs of positive affect (which, if the same criteria were applied as for negative behavioural signs, would presumably be calm acceptance of rider signals and delivery of the requested behavioural response).

**Table 4.** (a) Behaviour during ridden activities indicative of affective state (positive or negative), supporting evidence*, and (b) factors affecting this behaviour

4(a) BEHAVIOUR DURING RIDDEN ACTIVITIES				
Behavioural Events	Signs of positive affective state	Basis of justification	Signs of negative affective state	Basis of justification
Ear and mouth position / movement	Neutral ear position	+ve SIT (Thorbergson *et al*. 2016)	Ears back	PS (Kaiser *et al*. 2006; McDuffee *et al*. 2022) –ve SIT (Thorbergson *et al*. 2016)
		PS (Ruet *et al*. 2020)	Lip movements, teeth grinding	PHYS, –ve SIT (Visser *et al.* 2009)
	Ears forward		Abnormal mouth movements	PS, AVO (Ruet *et al*. 2020)
	Playing with bit	PHYS, +ve SIT (Mendonça *et al.* 2020)	Chewing, mouth opening	–ve SIT (Quick & Warren–Smith 2009)
Head / neck position and movements	Low head	+ve SIT (Thorbergson *et al*. 2016)	Head tossing, shaking, turning	PREF (von Borstel *et al*. 2009); PHYS, AVO (Christensen *et al*. 2014); PS (Kaiser *et al.* 2006; Ruet *et al*. 2020; Christensen *et al.* 2021)
			High head carriage,	PHYS, –ve SIT (Visser *et al.* 2009)
			Looking around	PHYS (Mendonça *et al*. 2020)
			Pulling at reins	–ve SIT, AVO (Thorbergson *et al.* 2016)
			Extended head and neck angle	–ve SIT (Robinson & Bye 2021)
Body and leg movements			Body tension	PHYS, –ve SIT (Visser *et al.* 2009)
			Reluctance to move forwards	PS, AVO (Ruet *et al.* 2020)
			Attempted bucks	AVO, PREF (von Borstel *et al.* 2009)
			Pawing at ground	–ve SIT, AVO (Quick & Warren–Smith 2009)
			Shying, bolting	PS, AVO (Christensen *et al.* 2021)
			Reduced carpal flexion	PS, –ve SIT (Robinson & Bye 2021)
Tail movements			Tail swishing	PS, PREF (von Borstel *et al*. 2009); PS (Christensen *et al.* 2021; McDuffee *et al*. 2022); –ve SIT (Quick & Warren–Smith 2009; Thorbergson *et al*. 2016); PS, –ve SIT (Ruet *et al.* 2020)
Vocalisations	Snort	+ve SIT (Stomp *et al*. 2020)		
Defaecation			Increased occurrence	PS, PHYS (Kaiser *et al*. 2006)

4(b) FACTORS AFFECTING BEHAVIOUR		
Home environment	Behaviour in the home environment (related to the type of home environment, see Table 2[a] and [b]) was found to reflect behaviour when ridden. Horses that were unresponsive to the environment in the stable were also more reluctant to move forwards when ridden, those showing stereotypical behaviour, aggression towards humans or hypervigilance in the stable displayed more negative behaviours/signs of discomfort when ridden (including head shaking/tossing, abnormal mouth movements, ears back and tail swishing).	(Ruet *et al.* 2020)
	Rider assessed ‘willingness to perform’ was scored lowest in those horses with no turn–out.	(Werhahn *et al.* 2012)
Training method	Sympathetic training method resulted in reduced negative behavioural signs as compared with conventional training method.	(Visser *et al.* 2009)
Rider	The rider was shown to be the main factor associated with conflict behaviour in ridden horses.	(Christensen *et al*. 2021)
	In a therapeutic riding session, ‘at–risk’ children were associated with an increase in the stress–related behaviours exhibited by the horse.	(Kaiser *et al.* 2006)
	Increased signs of stress were displayed by the horse during mounting and dismounting in both therapeutic riding sessions, and across the wider equine sector.	(McDuffee *et al*. 2022; Dyson *et al*. 2022)
	Wither scratching after a ridden session resulted in behavioural signs of relaxation when compared with neck patting or no interaction for one minute (while still mounted).	(Thorbergson *et al*. 2016)
Position of head/neck	Coercively obtained hyperflexion of the head and neck resulted in increased signs of discomfort and fear (and increased rein tension).	(von Borstel *et al.* 2009; Christensen *et al*. 2014)
Tack	Bitted vs bitless bridles:	
	Horses undergoing foundation training (bridling, long reining, and riding) wearing a bitless bridle exhibited less chewing, opening of the mouth, pawing the ground, and tail swishing throughout all stages and more head lowering during long reining than with the bitted bridle.	(Quick & Warren–Smith 2009)
	The pressure exerted on the facial structures when a bitless bridle was used was shown to affect locomotion and could be detrimental to the horse.	(Robinson & Bye 2021)
	Tack in general: Avoidance/abnormal behaviours were common during tacking up.	(Dyson *et al*. 2022)
Type of exercise	Including lateral exercises (shoulder in) in ridden work reduced ‘distracted’ behaviour and increased relaxation in the horse.	(Mendonça *et al*. 2020)
Stage of training	As training progresses, fewer negative behavioural signs occur.	(Quick & Warren–Smith 2009)
Weather	Ambient temperature and wind speed affected the response of the horse to the rider. Increased lethargy/reduced willingness to work was associated with ambient temperatures > 26°C, and over–active behaviour with a reduced response to the rider occurring when wind speeds were above 5.5 m s^–1^.	(Janczarek *et al.* 2015)
Links with performance	A positive relationship was found between the frequency of behaviours indicative of conflict (head shaking, tail swishing, bucking, and bolting), and faults accrued during a showjumping competition.	(Jastrzȩbska *et al*. 2017)

* Key to supporting evidence: Past studies (PS), physiological measures (PHYS), assumption of associated pain (PIP), situations deemed positive or negative (+ve/-ve SIT), positive or negative social interactions (horse/human) (+ve/-ve SOC), approach/avoidance (APP/AVO), choice (PREF).

The most common justification for assigning valence to the behaviours recorded was reference to previous studies and to commonly accepted interpretations of the responses to rider signals during different ridden scenarios. Of the sources cited as informing the ethograms used, only five were cited by more than one study. McDonnell (2003) and Waring (2003) were each cited by three separate studies, and Young *et al.* (2012), McGreevy *et al.* (2005), and Dyson *et al.* (2018) were each cited by two separate studies (although the latter was only cited to support further studies by the same research group).

Ruet *et al.* (2020) compared ridden performance with behavioural indicators of welfare state in the home environment, using both objective behavioural assessment and the more subjective qualitative behaviour assessment (QBA). They found an association between horses recorded as hypervigilant and/or performing stereotypical behaviours in the stable, and both signs they attributed to negative affective state when ridden (ear and tail positions) and QBA scores (Ruet *et al.* 2020). Out of the eleven behavioural and postural indicators used to assess affective state when ridden, only three could potentially be considered as positive signs (ears forwards or asymmetric, and snorts). Within the QBA list of thirteen descriptors, six could be described as positive in some scenarios (at ease, curious, friendly, happy, looking for contact, relaxed), although may not be appropriate for assessing ridden behaviour. No association between behaviour in the ridden test and QBA scores was reported (Ruet *et al.* 2020).

The challenges associated with attributing affective state to specific behaviours were highlighted by von Borstel *et al.* (2009) where the inclusion of the vocalisation termed a ‘snort’ as a negative sign was supported by Waring (2003) but deemed a positive sign by Fraser (1998). Similarly, Visser *et al.* (2009) interpreted snorts as negative signs in contrast to Stomp *et al.* (2020). It is possible that subtle differences in acoustic structure relate to valence, but these may be hard (or impossible) for the human ear to distinguish. More conclusive evidence of the subjective experience of the horse when ridden was provided by von Borstel *et al.* (2009) in the form of a choice test. Horses showed a preference for an area where they had not been ridden in a coercively obtained head/neck position, with the latter hyperflexion also being found to be associated with increased fear-related responses (von Borstel *et al.* 2009).

Physiological measures were recorded in ten of the retained studies. Christensen *et al.* (2021) found increases in HR and salivary cortisol concentration associated with specific riders. Visser *et al.* (2009) recorded higher HR in horses that had been trained using conventional as opposed to more sympathetic methods in the final ridden test, although there was no difference in behaviour or performance. When comparing competitive success in showjumping with levels of salivary cortisol and occurrences of conflict behaviour, increased levels of both were associated with poorer performance (Jastrzębska *et al.* 2017). Whether such differences relate to physical exertion and/or fitness, or whether they reflect ‘mental stress’, is questionable. It has been noted previously that although physiological measures provide information about arousal levels, the extent to which they can be used to determine the valence of responses has yet to be determined, particularly in scenarios involving physical exertion (Hall *et al.* 2018).

It has been demonstrated that it is possible to adopt a responsive approach to the management procedure of grooming, which makes it a positive experience for the horse (Lansade *et al.* 2019). A similar more responsive approach to other management procedures, making them less aversive and pleasurable where possible, would reduce the cumulative effect of negative experiences for the horse. The same applies to ridden work where the rider was found to be the main factor associated with signs of conflict in the horse (Christensen *et al.* 2021). Although the frequency of conflict behaviours has been associated with poor performance in showjumping (Jastrzębska *et al.* 2017), the occurrence of conflict behaviour was not found to relate to performance evaluation by dressage judges (Hamilton *et al.* 2022). In addition to recommending further training for riders, adapting performance criteria to account for behavioural signs of negative affective state would be positive progress towards improved welfare for ridden horses.

The potential for using behavioural signs to assess affective state in this scenario is limited by the restraint/control exerted by the rider and/or equipment used in ridden work. Also, when horses are kept in constrained home environments (stables) any apparent enthusiasm for exercise when being ridden may be attributed to rebound behaviour. Consequently, behavioural signs of positive or negative anticipation during the preparation for ridden work may provide a more accurate means of assessment. Avoidance behaviours during tacking up and mounting, suggestive of negative anticipation, were found to be common in both leisure and sports horses by Dyson *et al.* (2022). Underlying pain or discomfort during ridden work must be eliminated as the potential cause of this behaviour before considering the need for re-training. Restrictive equipment, often used to mask ‘unwanted’ behaviours (for example, tight nosebands used to prevent mouth movement), undoubtedly causes discomfort, with rebound behaviours occurring once the restriction is removed (Fenner *et al.* 2016). Attempts to avoid the oral pressure of a bit (Eisersiö *et al.* 2023) and behaviour recorded when the horse’s body is forced and fixed into unnatural positions (Smiet *et al.* 2014) are further examples of how some aspects of ridden work and training can be very negative experiences for the horse. The impact of ridden work on the affective state of the horse can be assessed not only by ridden responses, but also by anticipatory and post-ridden behaviour. Improved welfare in ridden horses can only be achieved if their behavioural signs of confusion, discomfort or pain are responded to, not by restrictive equipment or harsher training methods, but by identifying the underlying cause.

Factors found to affect behaviour of the horse when ridden are shown in Table 4(b). Study details for the articles relating to this scenario are provided in Table S2.4 in the Supplementary material.

#### In summary

To date, no conclusive evidence has been presented to determine whether horses ever experience ridden activities as positive, or only neutral (responding as trained in response to rider signals) or negative. Where behaviour signifies pain or discomfort this must not be ignored. Developing an approach to training and all ridden activities (including equestrian sports) that has the potential to provide the horse with positive experiences is a challenge that must be addressed with urgency if equestrianism is to maintain its social licence to operate.

### Behaviour of the horse during non-procedural horse-human interactions

Horse-human interactions not related to specific management procedures or training were considered separately. The retained articles comprised thirteen in which factors that influence how horses respond to humans in general were investigated. Methods used to assess horse-human interactions included human approach tests, behavioural responses to visual, auditory, and olfactory signs of human emotion, and the effect of watching what were considered by the authors to be positive (grooming) or negative (veterinary intervention) horse-human interactions on behaviour. In the human approach tests the response to the human was assessed according to whether the horse behaved in a way that increased or decreased the distance between horse and human. Tests used included those that allowed the horse to freely choose whether to approach a stationary human (also termed a voluntary human approach test) (Sankey *et al.* 2010; Brubaker *et al.* 2021; Lerch *et al.* 2021) or those where the human approached the horse (forced human approach test) (Minero *et al.* 2018). Measures used to assess the response of horses (movement of horse in relation to the human and behavioural interactions with the human) in these tests are shown in Table 5(a)(i).

**Table 5.** (a) (i, ii, iii) Behaviour of the horse during non-procedural horse-human interactions indicative of affective state (positive or negative), (i) during behavioural tests, (ii) in response to human expressions of emotion, and (iii) to watching third party horse-human interactions, supporting evidence* (see footnote below table for an explanation of the abbreviations used), and (b) factors affecting this behaviour

5(a) BEHAVIOUR DURING HORSE-HUMAN INTERACTIONS				
	Signs of positive response	Source	Signs of negative response	Source
(i) Behavioural tests				
Voluntary human approach test	Approach human with ears forward, gaze towards human, interact with human (sniff, lick, nibble)	+ve SOC, APP (Lerch *et al.* 2021)	Increased latency to approach, greater distance maintained. Approach with ears back, threatening gaze, sniff, nibble, bite threat, kick threat.	–ve SOC, AVO (Lerch *et al.* 2021)
Forced human approach test	Horse remained still on human approach, sniffed human	+ve SOC, APP (Fureix *et al.* 2009)	Horse moves away from human approach	–ve SOC, AVO (Fureix *et al.* 2009)
(ii) Responses to human expressions of emotion				
	Responses to positive human expression	Source	Responses to negative human expression	Source
Visual: Facial expression			Angry facial expression: longer latency to approach, increased time spent looking, increased oral behaviour. Angry face: Left gaze bias.	–ve SOC, AVO (Merkies *et al.* 2022) –ve SOC, PHYS (Smith *et al.* 2016)
Body posture	Human submissive body posture encouraged approach	+ve SOC, APP (Smith *et al.* 2018b)	Disgust facial expression: Horse followed gaze less than with happy/neutral expression	–ve SOC (Baba *et al.* 2019)
Auditory	To human ‘laughter’: Right ear forwards bias	PS, +ve SOC (Smith *et al.* 2018a)	To human ‘growl’: Ears forward, immobile, freeze posture, vigilant	–ve SOC, AVO (Smith *et al.* 2018a)
Olfactory			To ‘fear’ body odour: head raised more frequently and for longer. Touched person more frequently and for longer.	–ve SOC (Sabniewicz *et al.* 2020)
(iii) Responses to watching third party horse–human interactions				
	Responses when watching video of positive interactions (grooming)	Source	Responses when watching video of negative interactions (veterinary intervention)	Source
	Increased grooming attempts (assistant/headcollar rope)	PHYS (Trosch *et al.* 2020)	High head and neck, ears forward, whites of eyes showing	PHYS (Trosch *et al.* 2020)

5(b) FACTORS AFFECTING BEHAVIOURAL RESPONSE TO HUMANS		
Home environment	Increased aggression shown towards humans by horses housed individually for long periods of time.	(Ruet *et al.* 2020)
	Reduced relaxation during human interactions shown by horses housed in individual housing (as opposed to those kept in pairs/groups).	(Harewood & McGowan 2005; Yarnell *et al.* 2015)
Individual human	Horses responded differently to the same type of facial expression (angry, sad, joy, neutral) produced by different human	(Merkies *et al.* 2022)
Experience/role of horse	In a voluntary human approach (VHA) test horses from a riding school background interacted more with the human than horses with experience of participation in equine assisted services (EAS).	(Lerch *et al.* 2021)
	During a grooming test, horses with experience of participation in EAS showed less affiliative behaviour towards the human than horses with no experience of EAS.	(Brubaker *et al.* 2021)
Forage provision	A positive association was found between the amount of forage fed and interaction with the human in a VHA test.	(Lerch *et al.* 2021)
Horse/pony	Ponies interacted less with the human in a VHA test than horses.	(Lerch *et al.* 2021)
Sex of horse	Geldings interacted more with the human in a VHA test than mares.	(Lerch *et al.* 2021)
Age/training	In one–year–old horses, humans approaching from the left side of the horse resulted in more negative responses (move away/threats) than when approaching from the right side. This laterality in response was not apparent in two–year–old horse that had undergone training.	(Sankey *et al.* 2011)

* Key to supporting evidence: Past studies (PS), physiological measures (PHYS), assumption of associated pain (PIP), situations deemed positive or negative (+ve/-ve SIT), positive or negative social interactions (horse/human) (+ve/-ve SOC), approach/avoidance (APP/AVO), choice (PREF).

The ability of horses to recognise signs of human emotion (visual, auditory, and olfactory) was demonstrated in eight studies. Responses to human facial expressions depicting positive and negative human emotions (including happy, angry, disgust, and neutral expressions) were recorded (from photographs: Smith *et al.* 2016; live: Baba *et al.* 2019; Merkies *et al.* 2022). The impact of facial expression, as well as non-verbal auditory stimuli (laughter, growling: Smith *et al.* 2018a), human body posture (Smith *et al.* 2018b) and human body odour (fear, non-fear: Sabiniewicz *et al.* 2020) on horse behaviour is shown in Table 5(a)(ii). Approach behaviour was more likely to occur in response to positive human facial expressions (Smith *et al.* 2016; Baba *et al.* 2019; Merkies *et al.* 2022), positive non-verbal communication (laughter: Smith *et al.* 2018a), and when the human adopted a submissive rather than a dominant body posture (Smith *et al.* 2018b). Human body odour associated with fear resulted in increased head raising and touching the human (Sabniewicz *et al.* 2020). Smith *et al.* (2016) recorded increased HR in response to the photographs of angry (as opposed to happy) human faces, whereas Merkies *et al.* (2022) found when using live actors (with facial expressions of anger, sadness, joy, and neutral) there was no effect on the horse’s HR.

Further evidence of the horse’s ability to recognise the valence associated with human expression was demonstrated in two studies investigating behavioural responses to incongruous cross-modal signals (facial expression and vocalisation: Nakamura *et al.* 2018; Trösch *et al.* 2019). In both studies, the horses reacted in accordance with the valence of the vocalisation, particularly if this was negative and incongruent with the visual image. Nakamura *et al.* (2018) recorded an increase in HR and length of time spent looking at the visual image if the negative vocalisation (scolding voice) was from a familiar human and incongruent with the facial expression on the image. Trösch *et al.* (2019) also recorded increased HR and attention paid to an incongruent image depicting facial expression when the valence of the human vocalisation was negative (phonetic sound representing anger). See also Part I of this review (Hall & Kay 2024; *Intra-species social behaviour*) where the salience of vocalisation as a means of communicating emotion between horses was discussed.

The potential for emotional contagion to occur when horses watched horse-human interactions on a screen was demonstrated by Trösch *et al.* (2020). Responses to watching interactions deemed positive (grooming) differed from those recorded when watching negative (veterinary procedures) horse-handler interactions, with contact-seeking behaviour and lower HR associated with the positive interactions. However, following the viewing, when presented with both handlers, increased contact-seeking behaviour was associated with the negatively interacting handler which the authors tentatively suggested could be linked to attempts at appeasement, but the reason was not clear.

What was evident from the findings of the retained articles was the ability of the horse to respond to human expressions of emotion, with preference generally given for positive signals. It is recognised that human emotion is contagious (Herrando & Constantinides 2021), and the attempted mutual grooming behaviour elicited by watching video footage of positive horse-human interactions during grooming (Trosch *et al.* 2020), suggests that emotional transfer can occur between human and horse (see Table 5[a [iii]).

Factors found to affect horse behaviour during non-procedural horse-human interactions are shown in Table 5(b).

Study details for the articles relating to this scenario are provided in Table S2.5 in the Supplementary material.

#### In summary

Given the evidence that horses respond to human expressions of emotion, a positive mood state while interacting with horses in whatever capacity may induce a more positive response from the horse and facilitate an improved horse-human relationship. Unpredictable or negative interactions with humans are likely to result in avoidance behaviour, or at least reduce the inclination for horses to approach humans. This may, in part, explain the lack of voluntary human interaction (Lerch *et al.* 2021) and reduced affiliative behaviour shown to humans (Brubaker *et al.* 2021) by horses with experience of participation in Equine Assisted Services (EAS) compared with those with no such experience.

## Behaviour of the horse during transport

The transport of domestic horses is a common occurrence for leisure, sporting, breeding, or veterinary purposes and is acknowledged as potentially having a negative impact on health and performance (Leadon 1994). Twelve articles relating to horse transport were retained, ten of which involved transport by road, with two involving air transport (Stewart *et al.* 2003; Munsters *et al.* 2013). It was acknowledged that there are physical demands associated with maintaining balance during road transport, and this was demonstrated by the results reported (for example, Waran & Cuddeford 1995; Padalino *et al.* 2012; Tateo *et al.* 2012; Padalino & Raidal 2020). Behaviour at different stages of transport was recorded, as well as the impact of factors such as space and position within the transport vehicle (Padalino *et al.* 2012; Padalino & Raidal 2020), and journey duration (Tateo *et al.* 2012). Behavioural measures taken during transport related to movement associated with maintaining balance (Waran & Cuddeford 1995; Padalino *et al.* 2012, 2018; Tateo *et al.* 2012; Padalino & Raidal 2020), behaviour attributed to signs of mental stress (Kay & Hall 2009; Padalino *et al.* 2018; Padalino & Raidal 2020) and other behavioural states including social interactions (Stewart *et al.* 2003; Knowles *et al.* 2010) and feeding (Waran & Cuddeford 1995; Stewart *et al.* 2003; Kay & Hall 2009). See Table 6(a) for behaviour associated with positive and negative responses to transport during and post-transportation.

**Table 6.** (a) Behaviour of the horse during and after transportation indicative of affective state (positive or negative), supporting evidence* (see footnote below table for an explanation of the abbreviations used), and (b) factors affecting this behaviour

6(a) BEHAVIOUR				
	Signs of positive affective state	Basis of justification	Signs of negative affective state	Basis of justification
Behavioural states (post–stressor – compared with pre–stressor)				
			Decreased time spent standing, stand resting and drinking post transport	–ve SIT (Ji *et al.* 2013)
			Feeding sooner when in stall post–transport	–ve SIT (Tateo *et al.* 2012)
Behavioural Events (while experiencing stressor)				
Head/neck movements	Decreased head tossing, head turning	PHYS, +ve SIT (Kay & Hall 2009)	Sniffing at ground	PHYS, –ve SIT (Knowles *et al.* 2010)
	Low head position	PS (Padalino *et al.* 2018)	Increased agonistic behaviour (ears back, biting)	–ve SIT, –ve SOC, AVO (Stewart *et al.* 2003)
			Avoidance behaviour (head turning)	
			Sniffing, licking, chewing	PS, –ve SIT (Padalino *et al.* 2018)
Body movements			Bracing posture	PHYS, –ve SIT (Waran & Cuddeford 1995; Padalino & Raidal 2020)
Leg/hoof movements	Decreased pawing	PHYS, +ve SIT (Kay & Hall 2009)	Increased pawing	PHYS, –ve SIT (Knowles *et al.* 2010)
			Left limb preference when stepping on ramp during loading	–ve SIT (Siniscalchi *et al.* 2014)
Vocalisations	Decreased vocalisation	PHYS, +ve SIT (Kay & Hall 2009)		
Feeding	Increased feeding	PHYS, +ve SIT (Kay & Hall 2009)	Decreased feeding	–ve SIT (Waran & Cuddeford 1995)

6(b) FACTORS AFFECTING BEHAVIOUR		SOURCE
Travelling companion	Travelling with a companion reduced behavioural and physiological signs of stress (and a surrogate companion – mirror – had similar but lesser effect)	(Kay & Hall 2009)
	Transport of semi–feral ponies singly caused behavioural signs of separation anxiety	(Knowles *et al.* 2010)
Stage of journey	Increased anxiety and left limb preference recorded when stepping onto loading ramp compared with stepping off.	(Siniscalchi *et al.* 2014)
	Stress–related behaviours occurred more frequently during the first hour of a journey by road (exploratory behaviour, sniffing, licking, chewing, and putting their nose outside their bay)	(Padalino *et al.* 2018)
	During air transport increased agonistic behaviour was recorded during ascent and descent	(Stewart *et al.* 2003)
Movement of vehicle	Vehicle movement during road transport resulted in bracing postures and behaviour to maintain balance.	(Waran & Cuddeford 1995; Padalino & Raidal 2020)
	Pawing and yawning were found to occur more when the vehicle was stationary than when moving.	(Padalino *et al.* 2018)
	A reduction in feeding behaviour was recorded while the vehicle was moving.	(Waran & Cuddeford 1995)
	Increases in heart rate were recorded during times of turbulence in air transport but not always associated with observed behaviour	(Munsters *et al.* 2013)
Post–transport rebound behaviour	Transportation is associated with confinement and restricted movement. Post–transport horses were found to move more and stand still less when compared with pre–transport behaviour.	(Ji *et al.* 2013)
	Feeding behaviour occurred soon after transportation.	(Tateo *et al.* 2012)
Behaviour and transport–related health issues	During road transport, time spent with head down was negatively associated with stress–related behaviours such as licking/chewing and pawing, and the latter were predictive of transport–related health issues.	(Padalino *et al.* 2018)

* Key to supporting evidence: Past studies (PS), physiological measures (PHYS), assumption of associated pain (PIP), situations deemed positive or negative (+ve/-ve SIT), positive or negative social interactions (horse/human) (+ve/-ve SOC), approach/avoidance (APP/AVO), choice (PREF).

### Pre-transport

It was reported that the loading phase of the journey was found to be the most stressful (Siniscalchi *et al.* 2014), with evasive and avoidance behaviour being used as a measure of this (Waran & Cuddeford 1995; Hendriksen *et al.* 2011; Siniscalchi *et al.* 2014). Increases in HR were recorded during loading into both road (Waran & Cuddeford 1995; Tateo *et al.* 2012; Siniscalchi *et al.* 2014) and air transport vehicles (Stewart *et al.* 2003; Munsters *et al.* 2013). Although Hendriksen *et al.* (2011) found that training to load horses using positive reinforcement resulted in a reduction in behavioural signs of discomfort (compared with using negative reinforcement), this was not associated with any difference in HR. Although the increases in HR may, in part, reflect increased arousal associated with anticipation (positive or negative), it is likely that the primary cause of this was the physical exertion involved in entering the vehicle.

### During transport

When the transport vehicle was moving horses spent more time in a braced position and leaning against the partition, and less time feeding than when the vehicle was stationary (Waran & Cuddeford 1995). Vehicle movement was associated with an increase in HR (Waran & Cuddeford 1995; Padalino & Raidal 2020), which was also recorded during air transport at times of take-off and landing, but not throughout the rest of the flight (Stewart *et al.* 2003; Munsters *et al.* 2013). Increases in HR were found to be reduced when the horse was transported with a live companion (as opposed to alone) (Kay & Hall 2009), or when the horse travelled with its head in a lowered position (Padalino *et al.* 2018). Although the latter finding may relate to improved balance when in this position, isolation stress during transport was shown to be a contributory factor (Kay & Hall 2009). In addition to a decrease in HR, travelling with a companion was also associated with a decrease in the behavioural signs of stress, including less time spent head tossing and vocalising, and more time spent eating than when travelling alone (Kay & Hall 2009).

### Post transport

Post-transport behaviour was recorded by Padalino *et al.* (2012), Tateo *et al.* (2012) and Ji *et al.* (2013). Following transportation for the first time (for 5 h, 15 mins), Przewalski horses (*Equus ferus prezwalskii*) moved more, stood resting less and drank less than pre-transport, and increases in faecal cortisol metabolites were recorded 24 h post-transport (returning to pre-transport levels within three days) (Ji *et al.* 2013). After three-hour road journeys an increase in feeding and drinking was recorded by Padalino *et al.* (2012), and by Tateo *et al.* (2012). The confinement and frequently unfamiliar environment associated with transport has an impact on behaviour both during and after a journey. Most stress-related behaviours were recorded during the first hour of a journey (Padalino *et al.* 2018) and journeys of over three hours resulted in more rebound behaviour following transport confinement than shorter journeys (Tateo *et al.* 2012).

See Table 6(b) for factors affecting horse behaviour during and post-transportation and Table S2.6 (Supplementary material) for details of the studies included in this scenario.

### In summary

All stages of this activity have the potential to negatively affect the horse, over and above the physical challenges of remaining upright. Careful pre-planning, training, vehicle design and providing a travel companion, as well as allowing sufficient recovery time should all be travelling requirements. The fact that evasive and avoidance behaviour is so prevalent at the loading stage is evidence that many horses are at best apprehensive about entering confined vehicle spaces, particularly during initial training, and that measures that can be taken to ameliorate this should be applied.

### Behaviour of the horse during training other than when ridden (methods and equipment)

Nine articles with a focus on training and groundwork, which did not include riding the horse, were retained. These included behavioural responses to foundation training (Nittynen *et al.* 2022), traditional compared with natural horsemanship training (Fureix *et al.* 2009), responses associated with positively or negatively reinforced training (Sankey *et al.* 2010; Larssen & Roth 2022), different head and neck positions during lunging (Smiet *et al.* 2014), a low-intensity, stress-provoking task (walking backwards: Rietmann *et al.* 2004b) and the effect of restrictive and/or pressure-inducing equipment (tight nosebands: Fenner *et al.* 2016; rein tension/bit pressure: Eisersiö *et al.* 2021, 2023). The measures used either focused on behavioural responses towards humans, reactivity in novel object and isolation tests, and signs of discomfort and/or rebound behaviour. Behaviours signifying positive and negative responses to training other than when ridden are shown in Table 7(a).

**Table 7.** (a) Behaviour of the horse during training other than when ridden indicative of affective state (positive or negative), supporting evidence* (see footnote below Table for an explanation of the abbreviations used), and (b) factors affecting this behaviour

7(a) BEHAVIOUR				
Behavioural Events	Signs of positive affective state	Basis of justification	Signs of negative affective state	Basis of justification
Ear, nose, and mouth position/movement	Ears forward/moving	PHYS (Smiet *et al.* 2014)	Wide open nostrils, teeth grinding	PHYS (Smiet *et al.* 2014)
			Ears laid back	PHYS, –ve SIT (Sankey *et al.* 2010)
			Chewing, mouth opening	–ve SIT (Eisersiö *et al.* 2023)
			Rebound behaviour following release of noseband pressure (increased yawning, swallowing, licking)	PHYS (Fenner *et al.* 2016)
Head/neck position and movement	Affiliative interactions with human: touching with muzzle, lips, teeth. Exploratory behaviour: mouth, lips, muzzle, teeth.	PHYS (Niittynen *et al.* 2022) PHYS (Niittynen *et al.* 2022)	High head carriage	PHYS, –ve SIT (Rietmann *et al.* 2004b) PHYS (Niittynen *et al.* 2022)
			Head tossing, tilting	PHYS (Smiet *et al.* 2014; Niittynen *et al.* 2022)
			Head movements	PHYS, –ve SIT (Sankey *et al.* 2010)
			Head lifting, head turned to right	PHYS, –ve SIT (Sankey *et al.* 2010)
Body and leg movements	Increased interest/approaches human handler and remains close	PHYS, +ve SIT (Sankey *et al.* 2010)	Attempts to avoid task (stopping, moving backwards, turning, rearing)	PHYS, –ve SIT (Rietmann *et al.* 2004b)
			Slowing gait, moving backwards	PHYS (Niittynen *et al.* 2022)
			Slow to approach human handler, stepping sideways to avoid	PHYS (Smiet *et al.* 2014)
			Freeze (tense immobility)	PHYS, –ve SIT (Sankey *et al.* 2010)
			Sudden acceleration (forwards/sideways)	PHYS (Niittynen *et al.* 2022)
Tail movements			Tail swishing	–ve SIT (Rietmann *et al.* 2004b)
Vocalisations			Snorting	PHYS (Niittynen *et al.* 2022)

7(b) FACTORS AFFECTING BEHAVIOURAL RESPONSES TO TRAINING		
Physiological/early experiences	Positive correlation between salivary oxytocin and affiliative human-directed behaviours during foundation training in young horses (yearlings, two and three-year-olds)	(Niittynen *et al.* 2022)
Type of reinforcement	Increased positive interest in human interaction following positive reinforcement training; increased aggression/threats following negative reinforcement.	(Sankey *et al.* 2010; Larrsen & Roth 2022)
	Correct/timely application of pressure release (negative reinforcement) promotes a faster response, which also results from a reduced signal from the trainer.	(Eisersiö *et al.* 2021)
Pressure from equipment	Head, neck, mouth movements associated with attempts to avoid oral pressure from the bit (raising head, opening mouth, biting on the bit).	(Eisersiö *et al.* 2023)
	Restricted behaviour resulting from noseband tightness increased following pressure release (yawning, swallowing, licking)	(Fenner *et al.* 2016)
Restricting head/neck position/movement	Restricting the position of the head/neck (both in a raised and flexed position) causes behavioural and physiological stress responses, including conflict behaviour.	(Smiet *et al.* 2014)

* Key to supporting evidence: Past studies (PS), physiological measures (PHYS), assumption of associated pain (PIP), situations deemed positive or negative (+ve/-ve SIT), positive or negative social interactions (horse/human) (+ve/-ve SOC), approach/avoidance (APP/AVO), choice (PREF).

Following positive reinforcement training, increased approach/contact-seeking behaviour towards humans was recorded (Sankey *et al.* 2010; Larssen & Roth 2022), which may in part be the association of the human with food as this was used as the primary positive reinforcer during training. An association between an increase in HR and negative as opposed to positive reinforcement was found by Sankey *et al.* (2010), but reinforcement type was not associated with differences in hair cortisol levels (Larssen & Roth 2022). However, the type of reinforcement used had no effect on overall affective state as determined by judgement bias testing (Larsson & Roth 2022).

Fureix *et al.* (2009) found reduced latency to approach a motionless human in horses trained using natural horsemanship methods as opposed to traditional training, but although reduced responses to isolation and novel object tests occurred following training, this did not differ with training type (Fureix *et al.* 2009). As well as differences in response associated with the type/approach to training, a variation in how young horses respond to training was found by Nittynen *et al.* (2022). During foundation training, increases in salivary oxytocin occurred in horses showing affiliative, human-directed behaviours, with decreases in salivary oxytocin occurring in those showing signs of discomfort (Nittynen *et al.* 2022). The authors suggest that salivary oxytocin may be a useful non-invasive indicator of the subjective experience of training on young horses (Nittynen *et al.* 2022). In the same study, decreases in salivary cortisol concentration were recorded as the training progressed, alongside decreased signs of fear and discomfort, but the former increased with longer training sessions, suggestive of a link with physical exertion (Nittynen *et al.* 2022). Horse-human interactions during training can undoubtedly have an impact on how horses perceive humans, but it is unclear the extent to which this contributes to more general affective state.

The exercise of backing up in hand was shown to be challenging for horses by Rietmann *et al.* (2004b), who found correlations between HR and HRV parameters and behaviour during this low intensity but mentally stressful exercise (head high, explosive deviation and stopping). Backing up was also used to investigate the relationship between rein tension and behaviour, and the effect of a bit during in-hand training (Eisersiö *et al.* 2021, 2023). When a bit was used during the exercise (rather than a bit-less halter), an increase in head and neck movements, and oral behaviours was recorded (Eisersiö *et al.* 2021). Specific behaviours were found to affect rein tension, with increases of tension occurring with head movement down and forwards, decreased tension when the head was raised, the mouth open, and/or biting on the bit (Eisersiö *et al.* 2023). Clear behavioural signs linked with attempts to relieve bit pressure on the mouth were recorded. It was also demonstrated that the correct application of negative reinforcement enabled rapid decreases in the rein pressure required to train the task (Eisersiö *et al.* 2021). Human training in the correct application of learning theory and an awareness of the behavioural signs of discomfort in the horse would both be beneficial in reducing the negative impact of equestrianism on horse welfare.

Restricting movement during training both masks the response of the horse and is likely to cause discomfort. Lunging horses with their head and necks maintained in a specific position (variations in elevation and flexion) resulted in attempted avoidance behaviour when the side reins were being attached and stepping backwards when fitted to maintain an elevated head position with the bridge of the nose around the vertical (Smiet *et al.* 2014). This latter position also resulted in the most conflict behaviour and increases in salivary cortisol concentration. Negative anticipatory behaviour was also recorded when side reins were fixed to maintain the position with the neck lowered and flexed, and the bridge of the nose was pointing towards the carpus (Smiet *et al.* 2014). The impact of noseband tightness on physiology and behaviour while standing in a test area for ten minutes was assessed by Fenner *et al.* (2016). In the tight noseband condition, an increase in HR, decreased HRV and an increase in eye temperature were recorded. Following the release of the pressure when the noseband was unfastened, yawning, swallowing, and licking (rebound behaviours) occurred (Fenner *et al.* 2016). There is clear evidence that movement restriction is at best unnecessary during training, unhelpful in that it reduces the feedback that the horse can give to the human trainer, and at worst a negative and potentially painful experience for the horse.

Factors affecting behavioural responses during training other than when ridden and in response to the equipment used are shown in Table 7(b). See Table S2.7 in the Supplementary material.

#### In summary

All approaches to training are likely to be experienced initially as challenging and potentially negative experiences for the horse. Evidence suggests that this lessens as training progresses, and the horse learns to make the association between behaviour and outcome. To ensure that this interaction with humans does not become an increasingly negative experience, the trainer must be consistent in the application of learning theory and both aware of, and responsive to, positive and negative behavioural responses from the horse. The use of equipment that restricts this behavioural expression will also restrict the ability of the trainer to respond appropriately and is likely to substantially increase the aversiveness of this interaction for the horse.

### General behavioural signs of affective state during interactions with humans

In the two-dimensional model of core affect proposed by Mendl *et al.* (2010), positive valence when the animal is in a relatively high state of arousal is associated with behaviour motivated by reward acquisition. In a comparative state of arousal, negative valence is associated with behaviour motivated by the avoidance of punishment (and potentially pain/discomfort). There were specific examples of avoidance behaviour in the scenarios reported in this review. In ridden work, behaviour associated with restrictive practices (von Borstel *et al.* 2009; Christensen *et al.* 2014) and behavioural signs of negative anticipation during preparation for being ridden (Dyson *et al.* 2022), again suggestive of avoidance attempts, should all be regarded as attempts by the horse to avoid negative consequences. During transport, avoidance behaviour was found to be prevalent at the loading stage (Waran & Cuddeford 1995; Hendriksen *et al.* 2011; Siniscalchi *et al.* 2014), potentially associated with the anticipation of entering an unknown environment and being separated from conspecifics. The occurrence of avoidance behaviour, in whatever context-specific form this takes, provides a clear signal that certain experiences are aversive to the horse. In contrast, examples of approach behaviour suggest that the horse anticipates a positive outcome. A tendency to approach a human displaying positive emotional signals as opposed to negative ones (for example, Smith *et al.* 2018a,b; Merkies *et al.* 2022), suggests both the ability to recognise human expression and that this is used to predict a positive or negative outcome from subsequent interactions.

As well as providing context-specific information regarding the valence of emotional responses, approach/avoidance behaviour provides some insight into the horse’s overall affective state. Minero *et al.* (2018) found that horses assessed as being more at ease and relaxed in the home environment were most likely to readily approach a human when tested, and this behaviour in the home environment was judged to reflect a positive affective state. A positive association was found between the amount of forage provided and the tendency for horses to approach a human in a voluntary approach test (Lerch *et al.* 2021).

In states of low arousal, Mendl *et al.* (2010) describe the difference between positive and negative affective state as calm and relaxed or sad and depressed, respectively. It was noted in Part I of this review (Hall & Kay; *The home environment*) that differentiating between positive and negative signs when the horse is stationary may yield inconsistent conclusions, but general demeanour has the potential to provide an insight into the affective state of the horse. Hausberger *et al.* (2016) acknowledged the need for robust, objective measures of physical health, and used postural features and stance to differentiate between standing resting, standing observing, and standing withdrawn. Behavioural variability, as observed to increase when horses were recovering from low-level pain (Egan *et al.* 2021), which includes periods of alertness and relaxation, would appear to be one important positive sign. With the proviso that subjective assessment is likely to be influenced by human experience, training, and role within the equine sector (as found in ridden horses by Hall *et al.* 2014), a qualitative approach using evidence-based behavioural descriptors (similar to that used in the AWIN protocol: AWIN 2015), could be developed for wider application.

To enable an accurate response to the question posed by Dawkins (2004) regarding the health of the horse, behavioural signs of pain must be identified. Such signs should also determine the nature of subsequent interactions with humans, to ensure that these are not associated with increasing discomfort. Behavioural signs of negative anticipation may reflect underlying health issues or an association with past painful/negative experiences. Although it is not always easy to distinguish between behaviour indicative of pain and fear-related behaviour, and individuals vary in how both are expressed, this is an area that requires further investigation. Currently, pain-related behaviour may not always be interpreted as such, resulting in prolonged suffering, and potentially negatively affecting horse-human interactions.

Behaviour during many horse-human interactions is controlled/restricted by the human, and consequently signs indicative of positive or negative experiences may not be expressed by the horse and will also be affected by training. However, better recognition of behavioural signs of how horses experience interactions with humans would facilitate change in those identified as unpleasant for the horse.

### The challenges and limitations of this systematic review (Parts I [Hall & Kay 2024] and II combined)

The objectives of this systematic review, to identify evidence of how horse behaviour reflects affective state and its determining factors, with the overall aim of enabling the assessment of quality of life, were clear. However, assimilating the findings of the 179 disparate studies that were identified by the search terms and retained has proved to be complex. The variety of the scenarios explored reflects the diversity of activities and procedures that constitute the domestic life of the horse, at least in their role in sporting or leisure pursuits (as covered in this review). Notably, the findings suggest a complex inter-relationship between different aspects of the life of the domestic horse and the impact that they collectively have on their quality of life.

General challenges relating to research in horse welfare include accessibility/availability of subjects, consistency, and comparability of management conditions and ultimately the costs involved in studies involving the horse. Consequently, in many of the retained articles, low subject numbers are involved. A minimum for retention in this review was set at four subjects, although eight subjects per experimental group was suggested by Pongrácz (2023) where statistical analyses were performed. The articles retained in this review included fourteen with fewer than eight subjects per experimental group (seven subjects: Rogers *et al.* 2012; Smiet *et al.* 2014; Dalla Costa *et al.* 2017; Egan *et al.* 2021; six subjects: Harewood & McGowan 2005; Werhahn *et al.* 2012; Ji *et al.* 2013; Destrez *et al.* 2015; Reid *et al.* 2017; Carvalho *et al.* 2022; five subjects: Marliani *et al.* 2021; Robinson & Bye 2021; and four subjects: Quick & Warren-Smith 2009; McDuffee *et al.* 2022). Most of the studies with low subject numbers involved repeated measures, an exception being Quick and Warren-Smith (2009). In only two articles, both included in *Behavioural signs of pain in the horse*, were references made to power calculations to establish the minimum required sample size (Dodds *et al.* 2017; Ortolani *et al.* 2021). Dodds *et al.* (2017) noted that a power calculation was not carried out prior to the start of the study because there was no preliminary data upon which to base such analyses, and data collection was dependent upon the number of eligible horses that presented to the clinic during the study period. Similar challenges were noted by Ortolani *et al.* (2021) who reported calculating the required sample size as 24 but ended up with only 23 subjects.

At least in part, it is the result of challenges relating to availability, controllability, and cost that this review is based on multiple, relatively small studies. It does however provide a broad overview of what is also a large and varied sector. However, one of the downfalls of the number of articles retained within this review has been the time that it has taken to evaluate and assimilate the results of 179 separate studies. The time lapse between the date of the search deadline and manuscript submission has undoubtedly resulted in the omission of the most recent articles, but those retained provide robust evidence of measures that must be taken to improve the quality of life of the domestic horse.

Within the retained articles only three examples of studies where preference tests had been included were identified (see Part I [Hall & Kay 2024]; Table 2(b): preferred method of forage presentation in horse groups, Melvin *et al.* 2020; Part I (Hall & Kay 2024); Table 3(b): preference for companion over food in mares, Górecka-Bruzda *et al.* 2022; and Part II; *Behaviour of the horse when ridden*: preference for a location associated with being ridden in a regular poll position as opposed to coercively obtained hyperflexion of the head and neck: von Borstel *et al.* 2009). Although constrained by human-selected options, further use of preference testing, and the comparison of behaviour in preferred and non-preferred situations could provide valuable insights into behavioural signs of positive affective state (with the proviso that individual differences in the behavioural expression of affective state will occur).

### Animal welfare implications

Once the behavioural needs of the horse have been addressed, the impact of other aspects of management and training require consideration. As evidenced by the number of scenarios covered in this review, and factors that have been shown to have a negative impact on behaviour, it is likely that these will have a negative effect on the QOL of the domestic horse. As acknowledged in other species (for example, experimental cattle and pigs; Ryan *et al.* 2021), QOL is determined by the cumulative impact of past experiences, and the balance of pleasant and unpleasant experiences that horses have in their many and varied interactions with humans will contribute to their QOL. Whenever possible, management procedures should be designed to reduce aversion and adapted to be pleasurable for the horse, as demonstrated during grooming by Lansade *et al.* (2018). Also, there should be careful consideration of whether potentially aversive procedures are necessary and where possible their duration and/or frequency reduced or avoided altogether. Adherence to the LIMA (least invasive, minimally aversive) principles, as included in the Joint Standards of Practice and Code of Ethics adopted by the International Association of Animal Behaviour Consultants (IAABC) in 2018 (https://iaabc.org/code-of-ethics), should be used to guide all aspects of horse management and training. Clearly, some necessary procedures may be unavoidably aversive (for example, veterinary procedures) but any negative impact can be reduced by ensuring that the human handler is skilled in the procedure and that it is completed as quickly as possible (Górecka-Bruzda *et al.* 2017). Familiarity with the handler (Marsbøll & Christensen 2015; Liehrmann *et al.* 2022), physical restraint (Guinnefollau *et al.* 2021), the environment in which the procedure is carried out and the level of training/habituation to the procedure, will all affect the experience of the horse. The potentially negative cumulative impact of repeated aversive procedures (as may be required in a veterinary context) must be acknowledged and measures taken to provide positive experiences to counterbalance this.

The extent to which these recommendations are applied will ultimately depend upon the decisions and behaviour of those responsible for caring for the horse. In agreement with the first principle of humane livestock farming compiled by The Council on Animal Affairs in The Netherlands, there must be a recognition of the intrinsic value of the animal as a sentient being that can experience pain and pleasure (Council on Animal Affairs 2021). Acknowledgement of the importance of providing the horse with sufficient opportunities to fulfil its natural behavioural needs, and recognition of the inextricable link between behavioural satisfaction and affective state, is the basis for moving closer to providing a good life for the domestic horse.

**Figure 1.**
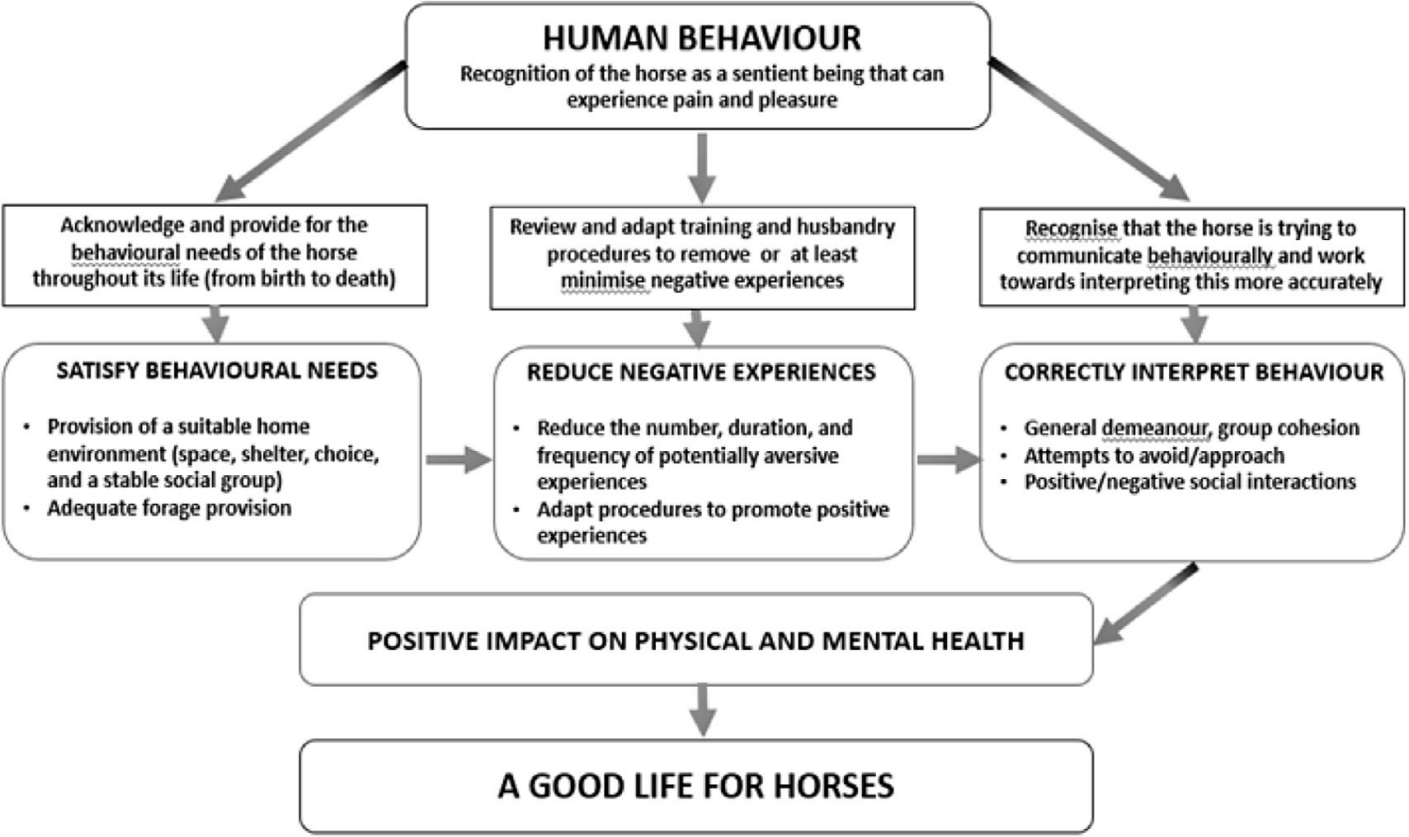
Flow chart demonstrating how human behaviour can facilitate the progression from satisfying the behavioural needs of the horse to providing a good life for the domestic horse.

To promote positive affective state in the domestic horse there are key issues that need addressing, as shown in Figure 1. The first challenge is for those within the equine sector to acknowledge that change is needed. Changes for the better, however small, in any of the areas identified, have the potential to make a positive impact on the horse. Once horse behaviour is recognised more clearly as an indicator of affective state, and subsequently results in a change in human behaviour, the horse will have a better chance of living a good life. The outcome for the horse is reliant upon these changes in human behaviour.

## Conclusion

Quality of life is determined by the balance between pleasant and unpleasant experiences over time and is reduced by the cumulative effect of repeated negative experiences. The role of the domestic horse as a sporting, leisure or companion animal is associated with many and varied interactions with humans. Behavioural signs indicative of whether these interactions are experienced as pleasant or unpleasant by the horse are confounded by human control and training and are open to misinterpretation. However, conclusions can be drawn in situations where there are opportunities for freedom of movement. Positive experiences of interactions with humans were associated with approach behaviour, negative ones with avoidance behaviour, but training could affect both. The absence of outward behavioural signs of attempted avoidance during interactions may result from training and/or restrictive practices, rather than reflecting the subjective experience of the horse. Many interactions with humans have the potential to be unpleasant for the horse and the equine sector now needs to consider what changes should be made to management and training procedures to make them less aversive and more pleasurable. A good life for horses is only possible if their species-specific needs are met and their lifetime experiences of interactions with humans are predominantly positive. The quality of life of the domestic horse can only improve if stakeholders from across the equine sector acknowledge the need for change and implement the findings of this review.

## Supporting information

Hall and Kay supplementary material Hall and Kay supplementary material

## Supplementary material

The supplementary material for this article can be found at http://doi.org/10.1017/awf.2024.41.
