# Supplementary material for: Living the good life? A systematic review of behavioural signs of affective state in the domestic horse (Equus caballus) and factors relating to quality of life. Part 2: Horse-human interactions
Source: Anim Welf. 2024 Oct 21;33:e41. doi: 10.1017/awf.2024.41 (PMC11514268; doi:10.1017/awf.2024.41)
Supplement: Hall and Kay supplementary material [file S0962728624000411sup001.pdf]

Supplementary material

Contents

1. Key to Tables S2.1-S2.7 (study design features)
2. Tables S2.1-S2.7 (with related reference lists of retained studies as referred to in each table)

1. Key to Tables S2.1-S2.7 (study design features)

Sex of subjects:	Mare (M), gelding (G), stallion (S), colt (C), filly (F)	
Study design:	Repeated measures (RM)<br>Between subjects (BS)<br>Testing associations (TA)<br>Observational (OB)	
Type of ethogram used to record behaviour:	E1 Specific behaviour recorded	Specific behavioural characteristic recorded and general 'emotion-related' ethogram not relevant (sound quality / vocalisation details, laterality, performance/ response to specific task, food sampling behaviour, personality assessment)
	E2 List of behaviours (no description)	a. List of behaviour with no reference to previous work<br>b. List of behaviour with reference to own previous work<br>c. List of behaviour with reference to other previous studies
	E3. Behaviour and descriptions	a. Full ethogram with no reference to previous work<br>b. Full ethogram with reference to own previous work<br>c. Full ethogram with reference to other previous studies
Supporting evidence presented to justify conclusions re: behaviour:	Reference to past studies (PS)<br>Physiological measures (PHYS)<br>Pain inducing procedures / evidence of pain (PIP)<br>Situation regarded as positive (+veSIT) or negative (-veSIT)<br>Positive (+veSOC) or negative (-veSOC) social interactions	

	Approach (APP) or avoidance (AVO) behaviour<br>Preferences tested / choice (PREF)
Specific physiological measures*:	Heart rate (HR), heart rate variability (HRV)<br>Blood pressure (BP)<br>Plasma cortisol (PC), salivary cortisol (SC), faecal cortisol metabolites (FC), hair cortisol (HC)<br>Eye temperature (ET), Ear-pinna temperature (EPT)<br>Rectal temperature (RT)<br>Skin surface temperature (ST)<br>Respiration rate (RR)<br>Salivary oxytocin (SOX)

* Other physiological measures specific to individual studies are not included in the tables below.

NA signifies not applicable.

2. Tables S2.1-S2.7 (with reference lists of retained studies as referred to in each table)

- S2.1 Behavioural signs of pain in the horse
- S2.2 Tests of emotional reactivity in the horse
- S2.3 Behaviour of the horse during handling and management procedures
- S2.4 Behaviour of the horse when ridden
- S2.5 Behaviour of the horse during non-procedural horse-human interactions
- S2.6 Behaviour of the horse during transport
- S2.7 Behaviour of the horse during training other than when ridden

Table S2.1. Study details for the retained articles (n = 22) focusing on behavioural signs of pain in the horse (see Section 1 above for the key to abbreviations used)

Study	Focus of study and other tests included	Study design	Length of study	Subject details			Behaviour recorded	Type of ethogram used	Supporting evidence	Physiological measures
				No.	Age	Sex				
Burn *et al.* 2010	General pain associated with poor health	TA	4 years	4504	Various	M,G,S	General alertness, human approach / contact.	E3b	PS, APP/AVO, signs of health	
Bussières *et al.* 2008	Scale - induced orthopaedic pain	BS		18	5-10yrs	M,G	Composite pain scale tested	E3a	PHYS	HR,RR,BP,RT,PC
Carvalho *et al.* 2022	Facial expression – low grade pain	BS		6	10-18yrs	M,G	Facial expression	E3c	Not found	ST
Dalla Costa *et al.* 2018	Facial expression – post castration	RM,OB		45	Not given	C	Horse grimace scale	E3b	PIP Pain related	
Destrez *et al.* 2015	Low forage diet	RM	10 weeks, 3 × 3-week trials (5-day gaps)	6	11-19yrs	G	Time budget Neophobia & sociability tests	Tests	Gut bacteria	
Dodds *et al.* 2017	General post-operative pain	BS,TA	6 months	36	1-21yrs	M,G,S	Personality Novel object & auditory test response	Personality	PIP Pain related	
Egan *et al.* 2021	Induced bilateral synovitis (intercarpal joints)	OB,TA	1 week	7	12-19yrs	M,G	Activity pattern Agitation	E3c	PIP Pain related	HR

Erber *et al.* 2012	Hot branding, microchipping	BS	2 days pre-procedure, 7 days post.	14	4-19 weeks	F,C	Behavioural response scored	E3c	PS, PHYS	HR,HRV,SC, ST
Fureix *et al.* 2010	Chronic back pain	TA	Exam then 5 tests	59	5-20yrs	M,G	Response to 5 x human approach / handling test	E2c	PIP Pain related	
Grint *et al.* 2017	Pressure testing – induced pain	BS		6 horses + 8 donkeys	Horses 3-4yrs	G	Anatomy and action of behavioural responses	E2a	PIP Pain related	
Kelemen *et al.* 2021	Lameness and recumbency	TA	Examination every 3 months	83	2-32yrs	M,G	Time budgets recorded automatically, lying down	Validation of activity monitor	PIP Pain related	
Laukkanen *et al.* 2023	Dental-related pain	TA	Dental exam and questionnaire	183	>5yrs	M,G, S	General, oral and feeding behaviour	E2c	PIP Pain related	
Lesimple *et al.* 2012	Chronic back pain	TA	Chiropractor and sEMG	18	10-26yrs	M,G, S	Neck posture	E2b	PIP Pain related	
Malmkvist *et al.* 2012	Gastric ulcers	BS	Endoscope then 1-2.5 wks later test on 2 consec. days	60	Average 7yrs	M,G, S	Response to novel object and postponed feeding tests	E3c	PIP Pain related, PHYS	HR,FC
Oliveira *et al.* 2022	Osteoarthritis and recumbency	TA	OB 1st 5 days in hospital	8	5-8yrs	M	Wakefulness, drowsiness, R/L lateral / sternal recumbency	E2a	PIP Pain related	

Ortolani *et al.* 2021	Ocular pain	BS	Pain scale development	23	Average 14yrs	M,G, S	General behaviour and ocular expression	E3c	PIP<br>PIP<br>Pain related	HR,RR,RT
Perron *et al.* 2023	Gastric ulcers	TA	Endoscope, 24 hrs, OB 3 consec. days	8	Average 15yrs	M,G	General and stress / pain related	E3c	PIP<br>Pain related<br>PHYS	HR,HRV
Pritchett *et al.* 2003	Colic surgery	BS	30hrs post recovery from anaesthesia	27	4-22yrs	M,G, S	Time budget Pain, posture & social numerical rating scale	E3a	PHYS (HR,PC)	HR,RR,PC
Reid *et al.* 2017	Anxiety or pain	RM	3 sessions over 2 days; 3hrs min. between sessions.	6	Average 12yrs	M	Anxiety and pain behaviour	E3c	PS,PHYS	HR,HRV
Rietmann *et al.* 2004	Laminitis	OB,TA	5 days at hospital	19	Average 13.6yrs	M,G	Clinical exam and weight shifting between contralateral limbs.	E3c single behaviour only	PS, pain level	HR,HRV,PC
Rochais *et al.* 2016a	Vertebral disorders	OB,TA	Back check and scan sampling	59,44	Not given	M,G	General and attention	E2c	PIP<br>Pain related	
Trindade *et al.* 2021	Post castration and anaesthesia	BS	24hrs pre- & post-anaesthesia	24	Average 4yrs, 9.5yrs	M,G, C	General, self-care, rest, discomfort, attention self/environ.	E3c	PIP<br>Pain related	

Table S2.1 References

Burn CC, Dennison TL and Whay HR 2010 Relationships between behaviour and health in working horses, donkeys, and mules in developing countries. *Applied Animal Behaviour Science* **126:** 109–118. https://doi.org/10.1016/j.applanim.2010.06.007

Bussières G, Jacques C, Lainay O, Beauchamp G, Leblond A, Cadoré JL, Desmaizières LM, Cuvelliez SG and Troncy E 2008 Development of a composite orthopaedic pain scale in horses. *Research in Veterinary Science* **85**: 294–306. https://doi.org/10.1016/j.rvsc.2007.10.011

Carvalho JRG, Trindade PHE, Conde G, Antonioli ML, Funnicelli MIG, Dias PP, Canola PA, Chinelatto MA and Ferraz GC 2022 Facial Expressions of Horses Using Weighted Multivariate Statistics for Assessment of Subtle Local Pain Induced by Polylactide-Based Polymers Implanted Subcutaneously. *Animals* **12**: 18. https://doi.org/10.3390/ani12182400

Dalla Costa ED, Pascuzzo R, Leach MC, Dai F, Lebelt D, Vantini S and Minero M 2018 Can grimace scales estimate the pain status in horses and mice? A statistical approach to identify a classifier. *PLoS ONE* **13:** 1–17. https://doi.org/10.1371/journal.pone.0200339

Destrez A, Grimm P, Cézilly F and Julliand V 2015 Changes of the hindgut microbiota due to high-starch diet can be associated with behavioral stress response in horses. *Physiology and Behavior* **149**: 159–164. https://doi.org/10.1016/j.physbeh.2015.05.039

Dodds L, Knight L, Allen K and Murrell J 2017 The effect of postsurgical pain on attentional processing in horses. *Veterinary Anaesthesia and Analgesia* **44**: 933–942. https://doi.org/10.1016/j.vaa.2016.07.010

Egan S, Kearney CM, Brama PAJ, Parnell AC and McGrath D 2021 Exploring stable-based behaviour and behaviour switching for the detection of bilateral pain in equines. Applied Animal Behaviour Science **235:** 105214. https://doi.org/10.1016/j.applanim.2021.105214

Erber R, Wulf M, Becker-Birck M, Kaps S, Aurich JE, Möstl E and Aurich, C 2012 Physiological and behavioural responses of young horses to hot iron branding and microchip implantation. *Veterinary Journal* **191:** 171–175. https://doi.org/10.1016/j.tvjl.2011.08.008

Fureix C, Menguy H and Hausberger M 2010 Partners with bad temper: Reject or cure? A study of chronic pain and aggression in horses. *PLoS ONE* **5:** 8. https://doi.org/10.1371/journal.pone.0012434

Grint NJ, Beths T, Yvorchuk-St Jean K, Whay HR and Murrell JC 2017 Analysis of Behaviors Observed During Mechanical Nociceptive Threshold Testing in Donkeys and Horses. *Journal of Equine Veterinary Science* **50**: 102–109. https://doi.org/10.1016/j.jevs.2016.11.001

Kelemen Z, Grimm H, Long M, Auer U and Jenner F 2021 Recumbency as an equine welfare indicator in geriatric horses and horses with chronic orthopaedic disease. *Animals* **11**: 1–16. https://doi.org/10.3390/ani11113189

Laukkanen T, Karma L, Virtala A-M, Mykkänen A, Pehkonen J, Rossi H, Tuomola K and Raekallio M 2023 Behavioral Signs Associated With Equine Cheek Tooth Findings. *Journal of Equine Veterinary Science* **121**: 104198. https://doi.org/10.1016/j.jevs.2022.104198

Lesimple C, Fureix C, de Margerie E, Sénèque E, Menguy H and Hausberger M 2012 Towards a Postural Indicator of Back Pain in Horses (*Equus caballus*). *PLoS ONE* **7: (**9). https://doi.org/10.1371/journal.pone.0044604

Malmkvist J, Poulsen JM, Luthersson N, Palme R, Christensen JW and Søndergaard E 2012 Behaviour and stress responses in horses with gastric ulceration. *Applied Animal Behaviour Science* **142**: 160–167. https://doi.org/10.1016/j.applanim.2012.10.002

Oliveira T, Santos A, Silva J, Trindade P, Yamada A, Jaramillo F, Silva L and Baccarin R 2022 Hospitalisation and Disease Severity Alter the Resting Pattern of Horses. *Journal of Equine Veterinary Science* **110**: 103832. https://doi.org/10.1016/j.jevs.2021.103832

Ortolani F, Scilimati N, Gialletti R, Menchetti L and Nannarone S 2021 Development and preliminary validation of a pain scale for ophthalmic pain in horses: The Equine Ophthalmic Pain Scale (EOPS). *Veterinary Journal* **278**: 105774. https://doi.org/10.1016/j.tvjl.2021.105774

Perron B, Ali ABA, Svagerko P and Vernon K 2023 The influence of severity of gastric ulceration on horse behavior and heart rate variability. *Journal of Veterinary Behavior* **59:** 25–29. https://doi.org/10.1016/j.jveb.2022.11.008

Pritchett LC, Ulibarri C, Roberts MC, Schneider RK and Sellon DC 2003 Identification of potential physiological and behavioral indicators of postoperative pain in horses after exploratory celiotomy for colic. *Applied Animal Behaviour Science* **80**: 31–43. https://doi.org/10.1016/S0168-1591(02)00205-8

Reid K, Rogers CW, Gronqvist G, Gee EK and Bolwell CF 2017 Anxiety and pain in horses measured by heart rate variability and behavior. *Journal of Veterinary Behavior: Clinical Applications and Research* **22**: 1–6. https://doi.org/10.1016/j.jveb.2017.09.002

Rietmann TR, Stauffacher M, Bernasconi P, Auer JA and Weishaupt MA 2004a The association between heart rate, heart rate variability, endocrine and behavioural pain measures in horses suffering from laminitis. *Journal of Veterinary Medicine Series A: Physiology Pathology Clinical Medicine* **51**: 218–225. https://doi.org/10.1111/j.1439-0442.2004.00627.x

Rochais C, Fureix C, Lesimple C and Hausberger M 2016a Lower attention to daily environment: A novel cue for detecting chronic horses' back pain? *Scientific Reports* **6:** 1–7. https://doi.org/10.1038/srep20117

Trindade PHE, Taffarel MO and Luna SPL 2021 Spontaneous behaviors of post-orchiectomy pain in horses regardless of the effects of time of day, anesthesia, and analgesia. *Animals* **11(6):** 1–20. https://doi.org/10.3390/ani11061629

Table S2.2. Study details for the retained articles (n = 19) relating to tests of emotional reactivity in the horse (see section 1 above for the key to abbreviations used)

Study	Factors investigated in relation to reactivity	Study design	Length of study	Subject details			Behaviour recorded	Type of ethogram used	Supporting evidence	Physiological measures
				No.	Age	Sex				
Baragli *et al.* 2014	Age	TA	1 test per horse in stable	98	4-24yrs	M,G	Response to visual startle	E3c	PS,PHYS, -ve SIT	HRV
Baragli *et al.* 2021	Laterality	TA	1 test per horse in stable	77	4-24yrs	M,G	Lateral response to visual startle	E3c	PS, individual differences, -ve SIT	
Bulens *et al.* 2015	Age (& housing)	TA	10 mins/4 days pre-test, 1 test on 4 consec. days	54	2-22yrs	M,G	General behaviour in stable, response to novel object	E3c	Inconclusive	
De Boyer Des Roches *et al.* 2008	Laterality	TA	3 tests – 1 per day	38	5-21yrs	M	Lateral response to novel object	Visual fields	PS, -ve SIT	
Fortin *et al.* 2018	+ve / -ve context, learning and personality (fear)	BS	10 days – day 1 reactivity tests x6, 3-6 days context. 7-10 cond. in context. sit.	55	Average 7.9yrs	M	Personality Learning performance & behaviour in +ve,-ve, or neutral context	E2b	PS, learning performance in context, +ve/-ve SIT	HR,HRV,SC
Hausberger *et al.* 2011	Type of work (and response in reactivity tests)	TA	3 different reactivity	119	4-20yrs	G	Behavioural patterns / events in	E3c	PS, +ve/-ve SIT	

			tests once per horse				response to tests			
Lansade *et al.* 2008	Individual differences	RM	3-6 days habituation to test pen, 4 tests on 8 days (12-45)	110	8mnths-2.5yrs	F,C,G	Behavioural responses to fear eliciting stimuli	E2a	PS, -ve SIT	HR
Lansade *et al.* 2012	Isolation and emotional expression	BS	-1 day test & train 11 days isolation, 1-9 training, day 11 reactivity	25	Average 10 mnths	F,C	Learning and response to reactivity tests	E3b	PS, -ve SIT	
Larose *et al.* 2006	Laterality	TA	1 test per horse over 6 months	35	2-3yrs	F,C	Laterality and behavioural patterns in response to test	E3c	PS, -ve SIT	
Lee *et al.* 2021	Age, breed/type, sex	TA	1 test per horse	39	Average 9-13yrs	M,G	Behavioural score (escape response) & duration	E3c	PS,PHYS,APP, AVO	HR,HRV
Manrique et al. 2021	Individual differences (age)	RM	Habituation 30 mins, 2 min isolation	30	9-12 mnths	F,C	Isolation stress response	E3b	PS	HRV
Mendonça *et al.* 2019	Type of work (and novel test)	TA	Baseline data collection and 4 test situations on 1 day	41	Average 10.41yrs	M,G	Reward acquisition - exploratory, social interaction, latency to AP	E3c	PS,PHYS,APP, AVO	HR,HRV
Olczak *et al.* 2021	Fearfulness and learning	TA	3 months – 3 learning, 1 fear test	23	15-26 mnths	F,C	General behaviour, learning and	Methods followed	Duration nervous	HR,SC

			(over 1-5D, 3-4D in between)				response to fear test. Feed finish		behaviour PHYS (HR) (But effect of feeding?)	
Ricci-Bonot *et al.* 2021	Context and social buffering	RM	4 tests, 1 test per week	32	Average 13.5yrs	M,G	Reactivity scores (head up to escape)	E3b	PS,AVO,PHYS Context specific	HR
Rochais *et al.* 2016b	Withdrawn behaviour, auditory attention	TA	5 tests, 1 per day (5 consec. days)	27	Not given	M,G	Response to novel auditory stimuli	E2c	PS	
Rørvang & Christensen 2018	Attenuation of fear in company	TA	D habituated 2 weeks, 2 studies (both 2 tests over 2 days)	32x2	Adult / 9-16yrs, 2yrs	M	Scale of reaction (none to flight)	E3b	PS,AVO,PHYS	HR
Rørvang *et al.* 2021	Frustration when task unsolvable	RM	4 tasks	39	3-25yrs	M,G	Behaviour indicating frustration	E2b	PS, PHYS	HR
Safryghin *et al.* 2019	Individual differences	RM	2 tests (20 mins and 15 mins data) 1 in summer, 1 in autumn	20	6-18yrs	M,G	High / low arousal behaviour	E3c	PS, PHYS in context	HR
Squibb *et al.* 2018	Effect of training	TA	2 novel handing tests in one session	46	3-20yrs	M,G	Time to cross / pass stimulus and level of AVO movement	E2b	PS, AVO	HR,HRV,RT, ET

Table S2.2 References

Baragli P, Banti L, Vitale V and Sighieri C 2014 Effect of aging on behavioural and physiological responses to a stressful stimulus in horses (*Equus caballus*). *Behaviour* **151**: 1513–1533. https://doi.org/10.1163/1568539X-00003197

Baragli P, Scopa C, Felici M and Reddon AR 2021 Horses show individual level lateralisation when inspecting an unfamiliar and unexpected stimulus. *PLoS ONE*, **16**: 1–14. https://doi.org/10.1371/journal.pone.0255688

Bulens A, Sterken H, Van Beirendonck S, Van Thielen J and Driessen B 2015 The use of different objects during a novel object test in stabled horses. *Journal of Veterinary Behavior: Clinical Applications and Research* **10**: 54–58. https://doi.org/10.1016/j.jveb.2014.09.002

De Boyer Des Roches A, Richard-Yris MA., Henry S, Ezzaouïa M and Hausberger M 2008 Laterality and emotions: Visual laterality in the domestic horse (*Equus caballus*) differs with objects' emotional value. *Physiology and Behavior* **94**: 487–490. https://doi.org/10.1016/j.physbeh.2008.03.002

Fortin M, Valenchon M, Lévy F, Calandreau L, Arnould C and Lansade L 2018 Emotional state and personality influence cognitive flexibility in horses (*Equus caballus*). *Journal of Comparative Psychology* **132**: 130–140. https://doi.org/10.1037/com0000091

Hausberger M, Muller C and Lunel C 2011 Does work affect personality? A study in horses. *PLoS ONE* **6**(2). https://doi.org/10.1371/journal.pone.0014659

Lansade L, Bouissou MF and Erhard HW 2008 Fearfulness in horses: A temperament trait stable across time and situations. *Applied Animal Behaviour Science* **115**: 182–200. https://doi.org/10.1016/j.applanim.2008.06.011

Lansade L, Neveux C and Levy F 2012 A few days of social separation affects yearling horses' response to emotional reactivity tests and enhances learning performance. *Behavioural Processes* **91**: 94–102. https://doi.org/10.1016/j.beproc.2012.06.003

Larose C, Richard-Yris MA, Hausberger M and Rogers LJ 2006 Laterality of horses associated with emotionality in novel situations. *Laterality* **11(4):** 355–367. https://doi.org/10.1080/13576500600624221

Lee KE, Kim JG, Lee H and Kim BS 2021 Behavioral and cardiac responses in mature horses exposed to a novel object. *Journal of Animal Science and Technology* **63**: 651–661. https://doi.org/10.5187/jast.2021.e51

Manrique LP, Bánszegi O, Hudson R and Szenczi P 2021 Repeatable individual differences in behaviour and physiology in juvenile horses from an early age. *Applied Animal Behaviour Science* **235:** 105227. https://doi.org/10.1016/j.applanim.2021.105227

Mendonça T, Bienboire-Frosini C, Kowalczyk I, Leclercq J, Arroub S and Pageat P 2019 Equine activities influence horses' responses to different stimuli: Could this have an impact on equine welfare? *Animals* **9:** 1–14. https://doi.org/10.3390/ani9060290

Olczak K, Klocek C and Christensen JW 2021 Hucul horses' learning abilities in different learning tests and the association with behaviour, food motivation and fearfulness. *Applied Animal Behaviour Science* **245**: 105498. https://doi.org/10.1016/j.applanim.2021.105498

Ricci-Bonot C, Romero T, Nicol C and Mills D 2021 Social buffering in horses is influenced by context but not by the familiarity and habituation of a companion. *Scientific Reports* **11**: 1–10. https://doi.org/10.1038/s41598-021-88319-z

Rochais C, Henry S, Fureix C and Hausberger M 2016b Investigating attentional processes in depressive-like domestic horses (*Equus caballus*). *Behavioural Processes* **124:** 93–96. https://doi.org/10.1016/j.beproc.2015.12.010

Rørvang MV and Christensen JW 2018 Attenuation of fear through social transmission in groups of same and differently aged horses. *Applied Animal Behaviour Science* **209**: 41–46. https://doi.org/10.1016/j.applanim.2018.10.003

Rørvang MV, Ničová K, Sassner H and Nawroth C 2021 Horses' (*Equus caballus*) Ability to Solve Visible but Not Invisible Displacement Tasks Is Associated with Frustration Behavior and Heart Rate. *Frontiers in Behavioral Neuroscience* **15**: 1–13. https://doi.org/10.3389/fnbeh.2021.792035

Safryghin A, Hebesberger DV and Wascher CAF 2019 Testing for behavioral and physiological responses of domestic horses (*Equus caballus*) across different contexts - consistency over time and effects of context. *Frontiers in Psychology* **10**: 1–12. https://doi.org/10.3389/fpsyg.2019.00849

Squibb K, Griffin K, Favier R and Ijichi C 2018 Poker Face: Discrepancies in behaviour and affective states in horses during stressful handling procedures. *Applied Animal Behaviour Science* **202**: 34–38. https://doi.org/10.1016/j.applanim.2018.02.003

Table S2.3. Study details for the retained articles (n = 17) relating to behaviour of the horse during handling and management procedures (see Section 1 above for the key to abbreviations used)

Study	Focus of study and other tests included	Study design	Length of study	Subject details			Behaviour recorded	Type of ethogram used	Supporting evidence	Physiological measures
				No.	Age	Sex				
Ali *et al.* 2017	Lip twitching	RM	2 treatments (ear clipping)	8	Average 2.8yrs	M,G	Avoidance (head, body)	E2a	PHYS	HR,HRV
Birt *et al.* 2015	Flowtrition soft touch therapy	BS	60 mins x4 Av. 11 days between each session	14	Average 12yrs	G	Signs of stress / relaxation	E3c	PHYS (HR)	HR,ST
Dalla Costa *et al.* 2017	Horse Grimace Scale (HGS) / fear / emotion	RM	Av. 4 days habituation, 4 x test situs	7	4-30yrs	M,G, S	HGS	E3b	+ve / -ve SIT	
Durier *et al.* 2012	Early handling of neonates	BS	Treatment postpartum; test at 1 and 2 years	17	1-2yrs	F,C,G	Response to novel object (1yr) and isolation (2yr)	E2c E2b	-ve SIT	
Górecka-Bruzda *et al.* 2017	Aversiveness of husbandry procedures	RM	Pre-, during, post- 4x treatments	27	Pre-weaned foals	C,F	Frequency of avoidance behaviours	E3a	PHYS (HR) AVO	HR,HRV,SC
Guinnefollau *et al.* 2021	Responses during vet prac. sessions	RM	30 mins pre-class and practical session x3	20	Average 16yrs	M,G	Eating and behavioural events (stress indic.)	E3c	PS	HR
Hintze *et al.* 2016	Eye wrinkles during procedures	RM	Photo analysis	16	3-20yrs	M,S	Eyelid shape / eye features	E3c	+ve / -ve SIT	

Lansade *et al.* 2019	Horse emotion during grooming	OB	Av. Session 12 mins	69	Average 11.7yrs	M,G	Approach, relaxation / avoidance	E3b	APP / AVO	
Lansade *et al.* 2018	Type of grooming	BS	11 grooming sessions, follow-up 1yr	27	Not given	Not given	Approach / avoid; facial expression	E3a	APP / AVO, PHYS (OX)	HR,HRV,PC, OX
Liehrmann *et al.* 2022	Familiarity with human handler	TA	2 months, 4 x tests	76	2-26yrs	M,G	Reluctance to approach / walk over	E3a	APP / AVO	
Lelláková *et al.* 2021	Eye blinks during procedures, pre-post-police training	OB	At rest, during 2 x 2 procedures	9	6yrs	M,G	Eye blinks	E3c	-ve SIT<br>Not PHYS	SC
Marsbøll & Christensen 2015	Previous handling and fear tests	BS	Handling training and 3 x tests	24	3yrs	M,G	Behaviour in fear tests	E3a	APP / AVO	HR
McBride & Hemmings 2004	Massage at preferred sites	RM	7 days preparation, pre-, during, post data	10	8-20+yrs	M,G	+ve / -ve response to massage	E3a	APP / AVO, PHYS	HR
Padalino *et al.* 2019	Rug in full sun	BS	15 mins prep., 2 hours record	18	4-15yrs	M,G	Stress behaviours	E3c	PHYS	HR,RT, sweat
Schanz *et al.* 2019	Breed/type	TA	Photos	181	4 mnths – 28yrs	M,G, S	Eye wrinkle scale	E3b	Inconclusive	
Watson & McDonnell 2018	Handling interventions	BS	4 months data collection. Video scores	48	2-23yrs	M,G	Relaxation, avoidance, stress	E2b<br>E2c	AVO	HR
Whitaker *et al.* 2011	Anthelmintic administration	BS	Pre-, during, post, admin.	122	Various >1yr	M,G	Avoidance, stress	E2c	AVO, PHYS	HR

Table S2.3 References:

Ali ABA, Gutwein KL and Heleski CR 2017 Assessing the influence of upper lip twitching in naive horses during an aversive husbandry procedure (ear clipping). *Journal of Veterinary Behavior: Clinical Applications and Research* **21**: 20–25. https://doi.org/10.1016/j.jveb.2017.07.001

Birt MA, Guay K, Treiber K, Ramirez HR and Snyder D 2015 The Influence of a Soft Touch Therapy Flowtrition on Heart Rate, Surface Temperature, and Behavior in Horses. *Journal of Equine Veterinary Science* **35**: 636–644. https://doi.org/10.1016/j.jevs.2015.06.006

Dalla Costa E, Bracci D, Dai F, Lebelt D and Minero M 2017 Do Different Emotional States Affect the Horse Grimace Scale Score? A Pilot Study. *Journal of Equine Veterinary Science* **54**: 114–117. https://doi.org/10.1016/j.jevs.2017.03.221

Durier V, Henry S, Sankey C, Sizun J and Hausberger M 2012 Locomotor inhibition in adult horses faced to stressors: A single postpartum experience may be enough! *Frontiers in Psychology* **3:** 1–6. https://doi.org/10.3389/fpsyg.2012.00442

Górecka-Bruzda A, Jaworski Z, Suwała M, Sobczyńska M, Jastrzębska E, Ogłuszka M, Sankey C, Boroń M and Jezierski T 2017 Aversiveness of husbandry procedures for pre-weaned foals: A comparison using behavioural and physiological indices. *Applied Animal Behaviour Science* **191**: 31–38. https://doi.org/10.1016/j.applanim.2017.02.007

Guinnefollau L, Bolwell CF, Gee EK, Norman EJ and Rogers CW 2021 Horses' physiological and behavioural responses during undergraduate veterinary practical teaching classes. *Applied Animal Behaviour Science* **241:** May. https://doi.org/10.1016/j.applanim.2021.105371

Hintze S, Smith S, Patt A, Bachmann I and Würbel H 2016 Are eyes a mirror of the soul? What eye wrinkles reveal about a horse's emotional state. *PLoS ONE* **11:** 10. https://doi.org/10.1371/journal.pone.0164017

Lansade L, Bonneau C, Parias C and Biau S 2019 Horse's emotional state and rider safety during grooming practices, a field study. *Applied Animal Behaviour Science* **217**: 43–47. https://doi.org/10.1016/j.applanim.2019.04.017

Lansade L, Nowak R, Lainé AL, Leterrier C, Bonneau C, Parias C and Bertin A 2018 Facial expression and oxytocin as possible markers of positive emotions in horses. *Scientific Reports* **8**: 14680. https://doi.org/10.1038/s41598-018-32993-z

Lelláková M, Pavľak A, Lešková L, Florián M, Skurková L, Mesarčová L, Kottferová L, Takáčová D and Kottferová J 2021 Monitoring Blinks and Eyelid Twitches In Horses To Assess Stress During The Samples Collection Process. *Journal of Applied Animal Welfare Science* **26**: 530-539. https://doi.org/10.1080/10888705.2021.2008249

Liehrmann O, Viitanen A, Riihonen V, Alander E, Koski SE, Lummaa V and Lansade L 2022 Multiple handlers, several owner changes and short relationship lengths affect horses' responses to novel object tests. *Applied Animal Behaviour Science* **254**: 105709. https://doi.org/10.1016/j.applanim.2022.105709

Marsbøll AF and Christensen JW 2015 Effects of handling on fear reactions in young Icelandic horses. *Equine Veterinary Journal* **47**: 615–619. https://doi.org/10.1111/evj.12338

McBride SD, Hemmings A and Robinson K 2004 A Preliminary Study on the Effect of Massage to Reduce Stress in the Horse. *Journal of Equine Veterinary Science* **24**: 76–81. https://doi.org/10.1016/j.jevs.2004.01.014

Padalino B, Loy J, Hawson L and Randle H 2019 Effects of a light-colored cotton rug use on horse thermoregulation and behavior indicators of stress. *Journal of Veterinary Behavior* **29**: 134–139. https://doi.org/10.1016/j.jveb.2019.02.001

Schanz L, Krueger K and Hintze S 2019 Sex and age don’t matter, but breed type does-factors influencing eye wrinkle expression in horses. *Frontiers in Veterinary Science* **6**: 154. https://doi.org/10.3389/fvets.2019.00154

Watson JC and McDonnell SM 2018 Effects of three non-confrontational handling techniques on the behavior of horses during a simulated mildly aversive veterinary procedure. *Applied Animal Behaviour Science* **203**: 19–23. https://doi.org/10.1016/j.applanim.2018.02.007

Whitaker TC, Goupil X, Roy O, Marciat D and McGahie D 2011 Evaluation and comparison under field conditions of the stress response induced in horses when administered endoparasiticides in tablet or paste formulations. *International Journal of Applied Research in Veterinary Medicine* **9(1–2):** 6–14.

Table S2.4. Study details for the retained articles (n = 17) relating to behaviour of the horse when ridden (see section 1 above for the key to abbreviations used)

Study	Focus of study and other tests included	Study design	Length of study	Subject details			Behaviour recorded	Type of ethogram used	Supporting evidence	Physiological measures
				No.	Age	Sex				
Christensen *et al.* 2014	Hyperflexion	RM	3 test days, 10 minute ridden test per day	15	5-18yrs	M,G, S	Frequency & duration conflict behaviours	E3a	PS, PHYS (SC)	HR,HRV,SC
Christensen *et al.* 2021	Effect of rider	RM	10 days, 2xriders /per horse / day	10	6-13yrs	M,G	Evasive, conflict behaviours	E3b E3c	PS, PHYS	HR,SC
Dyson *et al.* 2022	Tacking up, mounting	OB	Convenience sampling (no time scale specified)	193	Adult	M,G, S	Responses to tacking up & mounting	E3b E3c	PS	
Dyson & Pollard 2021	Signs of pain / judges scores	OB	Competition footage 2018 -2020	147	Average 12yrs	M,G, S	Abnormal gait, signs of pain	E3b	PS	
Janczarek *et al.* 2015	Atmospheric conditions	TA	Daily data, for 2 months	16	10-12yrs	G	'Incorrect' ridden behaviour; mood, willingness	E2a	PHYS	HR,RT,RR
Jastrzębska *et al.* 2017	Show jumping. Transport / comp. stress	TA	Two rounds / horse at competitions	19	Average 11.3yrs	Not spec.	Conflict behaviour	E2a	PHYS, jumping faults	SC

Kaiser *et al.* 2006	Therapeutic riding	OB, RM	Sessions recorded over year	14	3-30yrs	M,G	Stress behaviours	E3c	PS	
Luz *et al.* 2021	Post-work rolling	OB	160 days	8	8-16yrs	M,G	Features of rolling	E3c	PS	
McDuffee *et al.* 2022	Therapeutic riding (mounting, dismounting)	RM	Up to 3x / week, 8 wks	4	8-22yrs	M,G	Stress behaviours	E3c	PS, PHYS	HRV,SC
Mendonça *et al.* 2020	Lateral work	RM	Two sessions (2 x 10 mins)	40	Average 11yrs	M,G, S	Looking around, bit play	E3a	PS, PHYS (HRV)	HR,HRV
Quick & Warren-Smith 2009	Bits/bitless	BS	Preparation & 11 days training	4	2yrs	M,G	'Unwanted' & stress behaviours	E2c	PS, PHYS	HR,HRV
Robinson & Bye 2021	Bridle pressure Bits/bitless	RM	30 mins x 3 consec. days	5	Average 14yrs	M,G	Head/neck position, locomotion	E2a	PS	
Ruet *et al.* 2020	Welfare state	TA	Standardised riding tests (approx. 8 mins) over 3 months	43	Average 12.8yrs	M,G	Behaviours associated with affect; QBA	E2a E3c	PS	
Stomp *et al.* 2020	Snorts	TA	Riding lessons 39-70 mins over 1.5 years	127	3-30yrs	M,G	Neck position, snorts	E3b	PS	
Thorbergson *et al.* 2016	Patting, wither scratching	RM	6 days	18	4-32yrs	M,G	Agitated, ambiguous, relaxed	E3a (refs cited in Intro.)	PS	HR,HRV
Visser *et al.* 2009	Training type	BS	5 weeks	28	Average 3.5yrs	M,G	Behaviour in human approach test	E3c	PS, PHYS (HR)	HR,HRV

							& training inc. ridden test			
von Borstel *et al.* 2009	Rollkur posture	RM, choice, fear test	Ridden maze session 30 mins & pref. test	15	6-23yrs	M,G	Stress behaviours, preference, fear stimulus	E3c	PS, PREF, PHYS	HR

Table S2.4 References:

Christensen JW, Beekmans M, van Dalum M and VanDierendonck M 2014 Effects of hyperflexion on acute stress responses in ridden dressage horses. *Physiology and Behavior* **128:** 39–45. https://doi.org/10.1016/j.physbeh.2014.01.024

Christensen JW, Munk R, Hawson L, Palme R, Larsen T, Egenvall A, König von Borstel UU and Rørvang MV 2021 Rider effects on horses' conflict behaviour, rein tension, physiological measures and rideability scores. *Applied Animal Behaviour Science* **234**: November 2020. https://doi.org/10.1016/j.applanim.2020.105184

Dyson S, Bondi A, Routh J, Pollard D, Preston T, McConnell C and Kydd JH 2022 An investigation of behaviour during tacking-up and mounting in ridden sports and leisure horses. *Equine Veterinary Education* **34**: e245–e257. https://doi.org/10.1111/eve.13432

Dyson S and Pollard D 2021 Application of the ridden horse pain ethogram to horses competing at the Hickstead-Rotterdam grand prix challenge and the British dressage grand prix national championship 2020 and comparison with world cup grand prix competitions. *Animals* **11**: 1–16. https://doi.org/10.3390/ani11061820

Janczarek I, Wilk I, Zalewska E and Bocian K 2015 Correlations between the behavior of recreational horses, the physiological parameters and summer atmospheric conditions. *Animal Science Journal* **86:** 721–728. https://doi.org/10.1111/asj.12343

Jastrzębska E, Wolska A, Minero M, Ogłuszka M, Earley B, Wejer J and Górecka-Bruzda A 2017 Conflict Behavior in Show Jumping Horses: A Field Study. *Journal of Equine Veterinary Science* **57:** 116–121. https://doi.org/10.1016/j.jevs.2017.07.009

Kaiser L, Heleski CR, Siegford J and Smith KA 2006 Stress-related behaviors among horses used in a therapeutic riding program. *Journal of the American Veterinary Medicine Association* **228**: 39–45. https://doi.org/10.2460/javma.228.1.39

Luz MPF, Maia CM, Gonçalvez HC and Puoli Filho JNP 2021 Influence of workload and weather conditions on rolling behaviour of horses and mules. *Behavioural Processes* **189:** 104433. https://doi.org/10.1016/j.beproc.2021.104433

McDuffee L, Carr L and Montelpare W 2022 An observational evaluation of stress in horses during therapeutic riding sessions. *Journal of Veterinary Behavior* **49:** 53–64. https://doi.org/10.1016/j.jveb.2021.11.009

Mendonça T, Bienboire-Frosini C, Sanchez N, Kowalczyk I, Teruel E, Descout E and Pageat P 2020 de la Guérinière was right: Shoulder-in is beneficial for the physical and mental states of horses. *Journal of Veterinary Behavior* **38**: 14–20. https://doi.org/10.1016/j.jveb.2020.05.003

Quick JS and Warren-Smith AK 2009 Preliminary investigations of horses' (*Equus caballus*) responses to different bridles during foundation training. *Journal of Veterinary Behavior: Clinical Applications and Research* **4:** 169–176. https://doi.org/10.1016/j.jveb.2008.12.001

Robinson N and Bye TL 2021 Noseband and poll pressures underneath bitted and bitless bridles and the effects on equine locomotion. *Journal of Veterinary Behavior* **44:** 18–24. https://doi.org/10.1016/j.jveb.2021.05.002

Ruet A, Biau S, Arnould C, Galloux P, Destrez A, Pycik E, Boichot L and Lansade L 2020 Horses Could Perceive Riding Differently Depending on the Way They Express Poor Welfare in the Stable. *Journal of Equine Veterinary Science* **94:** 103206. https://doi.org/10.1016/j.jevs.2020.103206

Stomp M, Masson A, Henry S, Hausberger M and Lesimple C 2020 Could snorts inform us on how horses perceive riding? *Behavioural Processes* **172**: 104041. https://doi.org/10.1016/j.beproc.2020.104041

Thorbergson ZW, Nielsen SG, Beaulieu RJ and Doyle RE 2016 Physiological and Behavioral Responses of Horses to Wither Scratching and Patting the Neck When Under Saddle. *Journal of Applied Animal Welfare Science* **19(3):** 245–259. https://doi.org/10.1080/10888705.2015.1130630

Visser EK, VanDierendonck M, Ellis AD, Rijksen C and Van Reenen CG 2009 A comparison of sympathetic and conventional training methods on responses to initial horse training. *The Veterinary Journal* **181(1):** 48–52. https://doi.org/10.1016/j.tvjl.2009.03.009

von Borstel UU, Duncan IJH, Shoveller AK, Merkies K, Keeling LJ and Millman ST 2009 Impact of riding in a coercively obtained Rollkur posture on welfare and fear of performance horses. *Applied Animal Behaviour Science* **116(2–4):** 228–236. https://doi.org/10.1016/j.applanim.2008.10.001

Table S2.5. Study details for the retained articles (n = 13) relating to the behaviour of the horse during non-procedural horse-human interactions (see section 1 above for the key to abbreviations used)

Study	Focus of study and other tests included	Study design	Length of study	Subject details			Behaviour recorded	Type of ethogram used	Supporting evidence	Physiological measures
				No.	Age	Sex				
Baba *et al.* 2019	Following human gaze	RM	1 week	14	3-24yrs	M,G	Turning nose in direction of human gaze (frequency & duration)	E3c	Behavioural response – gaze following, PS	
Brubaker *et al.* 2021	Horses with experience of EAS	BS	One day per horse (4 tests)	30	7-25yrs	M,G	Responses to human and temperament tests	E3c	APP / AVO, PS	
Lerch *et al.* 2021	Interest in humans (EAS/riding school)	BS	Recordings of 5 min tests collected over 10 years	172	4-29yrs	M,G	Voluntary +ve and -ve responses to human	E3b	APP/AVO, +veSOC, -veSOC, PS	
Merkies *et al.* 2022	Recognition human facial expression	RM	4 x one-minute tests per day, 2 days	20	6-25yrs	M,G	First look R/L eye, duration of gaze, approach	E3c	Laterality / duration of gaze	HR
Minero *et al.* 2018	QBA and HHI	TA	40 farm visits	355	Adult >5yrs	M,G	QBA and response to forced HAT	E3b	AVO	
Nakamura *et al.* 2018	Recognising human emotion (visual/auditory)	RM	Trials over 4 months	19	Average 14yrs	M,G	Response rate, total looking time	E3c	PHYS Gaze latency and duration	HR

Sabiniewicz *et al.* 2020	Recognition of human body odour	RM	3 test sessions in 1 month	21	2-9yrs	M,G, S	Response to human odour	E3c	APP / AVO +veSOC / -veSOC, PS	
Sankey *et al.* 2011	Training & side of human approach	BS	1-2 daily sessions, 1-2 hours	39	1 and 2yrs	M,G	Avoidance / affiliative response to human approach	E2c	APP / AVO +veSOC / -veSOC, PS	
Smith *et al.* 2016	Human facial expression	RM	2x2 sessions (2 months apart) over 10 months	28	4-23yrs	M,G	First look R/L eye, approach	E2a	PS, lateral bias, PHYS	HR
Smith *et al.* 2018a	Human non-verbal vocalisation	RM	Sessions over 7 months	32	Not spec.	Not spec.	Vigilant, avoidance / approach	E2a E2b	APP / AVO PS, lateral bias	
Smith *et al.* 2018b	Human posture	Choice test	Four test trials	30/ 45	7-26yrs	M,G	Choice of human, approach latency	E2a	APP, PREF, PS	
Trösch *et al.* 2020	Vicarious horse – human interactions	Mixed	Familiarisation, video projection and choice test per horse	47	Average 8.47yrs	M	Attention, stress response, choice	E3c E3b	APP, PS, PREF, PHYS	HR
Trösch *et al.* 2019	Visual / auditory human signals	Attention test	Familiarisation & 1 test session (6 trials)	34	Average 9.47	M	Gaze duration, vigilant, relaxed	E3b E3c	PS, PHYS Gaze APP/AVO	HR

Table S2.5 References

Baba C, Kawai M and Takimoto-Inose A 2019 Are horses (*Equus caballus*) sensitive to human emotional cues? *Animals* **9**: 9. https://doi.org/10.3390/ani9090630

Brubaker L., Schroeder, K., Sherwood, D., Stroud, D., & Udell, M. A. R. 2021 Horse behavior towards familiar and unfamiliar humans: Implications for equine-assisted services. *Animals* **11**: 8. https://doi.org/10.3390/ani11082369

Lerch N, Cirulli F, Rochais C, Lesimple C, Guilbaud E, Contalbrigo L, Borgi M, Grandgeorge M and Hausberger M 2021 Interest in humans: Comparisons between riding school lesson equids and assisted-intervention equids. *Animals* **11**: (9). https://doi.org/10.3390/ani11092533

Merkies K, Sudarenko Y and Hodder AJ 2022 Can Ponies (Equus Caballus) Distinguish Human Facial Expressions? *Animals* **12:** 2331. https://doi.org/10.3390/ani12182331

Minero M, Dalla Costa E, Dai F, Canali E, Barbieri S, Zanella A, Pascuzzo R and Wemelsfelder F 2018 Using qualitative behaviour assessment (QBA) to explore the emotional state of horses and its association with human-animal relationship. *Applied Animal Behaviour Science* **204:** 53-59. https://doi.org/10.1016/j.applanim.2018.04.008

Nakamura K, Takimoto-Inose A and Hasegawa T 2018 Cross-modal perception of human emotion in domestic horses (*Equus caballus*). *Scientific Reports* **8**: 1–9. https://doi.org/10.1038/s41598-018-26892-6

Sabiniewicz A, Tarnowska K, Świątek R, Sorokowski P and Laska M 2020 Olfactory-based interspecific recognition of human emotions: Horses (*Equus ferus caballus*) can recognize fear and happiness body odour from humans (*Homo sapiens*). *Applied Animal Behaviour Science* **230**: 105072. https://doi.org/10.1016/j.applanim.2020.105072

Sankey C, Henry S, Clouard C, Richard-Yris MA and Hausberger M 2011 Asymmetry of behavioral responses to a human approach in young naive vs. trained horses. *Physiology and Behavior* **104:** 464–468. https://doi.org/10.1016/j.physbeh.2011.05.009

Smith AV, Proops L, Grounds K, Wathan J and McComb K 2016 Horses give functionally relevant responses to human facial expressions of emotion (*Equus caballus*). *Biology Letters* **12(9):** 20150907. https://doi.org/10.1098/rsbl.2015.0907

Smith AV, Proops L, Grounds K, Wathan J, Scott SK and McComb K 2018a Domestic horses (*Equus caballus*) discriminate between negative and positive human nonverbal vocalisations. *Scientific Reports* **8(1):** 1–8. https://doi.org/10.1038/s41598-018-30777-z

Smith AV, Wilson C, McComb K and Proops L 2018b Domestic horses (*Equus caballus*) prefer to approach humans displaying a submissive body posture rather than a dominant body posture. *Animal Cognition* **21(2):** 307–312. https://doi.org/10.1007/s10071-017-1140-4

Trösch M, Cuzol F, Parias C, Calandreau L, Nowak R and Lansade L 2019 Horses categorize human emotions cross-modally based on facial expression and non-verbal vocalizations. *Animals* **9:** 862. https://doi.org/10.3390/ani9110862

Trösch M, Pellon S, Cuzol F, Parias C, Nowak R, Calandreau L and Lansade L 2020 Horses feel emotions when they watch positive and negative horse–human interactions in a video and transpose what they saw to real life. *Animal Cognition* **23:** 643-653. https://doi.org/10.1007/s10071-020-01369-0

Table S2.6. Study details for the retained articles (n = 12) relating to behaviour of the horse during transport (see section 1 above for the key to abbreviations used)

Study	Focus of study and other tests included	Study design	Length of study	Subject details			Behaviour recorded	Type of ethogram used	Supporting evidence	Physiological measures
				No.	Age	Sex				
Hendriksen *et al.* 2011	+ve/-ve reinforcement for trailer loading	BS	Training 3x week with at least 1 day between. 18 mins max – 15 sessions	12	7-20yrs	M,G	Discomfort (eyes, nostrils) and avoidance	E3c	PS	HR
Ji *et al.* 2013	Transport stress Przewalski horses	OB	22 hrs pre-transport (5hrs 15mins) 72hrs post-t	6	Adult	M,S	Behavioural states (& marking)	E3c	PS, PHYS	FC
Kay & Hall 2009	Isolation stress, mirror / companion	RM	3 x 30mins trailer journey	12	8-22yrs	M,G	Feeding, vocalisation, pawing, head turning /toss	E3a	PS, PHYS	HR,RT,EPT
Knowles *et al.* 2010	Transport stress semi-feral ponies	OB	Pre- / during 40.2-mile trip	145	<6mnths->25yrs	M,S, C,F	Handling scores (pre) Interactions, contact, movement	E3a	PS, -veSOC, PHYS	PC
Munsters *et al.* 2013	Transport by air	OB	Pre-, post- & 8-hour flight	9	Average 9.8yrs	M,G	Activity level scores	E3c	PS	HR,HRV
Padalino *et al.* 2012	Position during road transport	RM	Pre- 3-hour road	12	3-7yrs	M,G, S	Directional movement,	E2a	PS, PHYS	HR,RR,RT,PC

			journey, post +2 +4 hours				post-transp. behaviour			
Padalino & Raidal 2020	Effect of space, position during road transport	BS	12 hours road transport	26	4-20yrs	M	Balance and stress behaviours	E3c	PS, PHYS	PC
Padalino *et al.* 2018	Transport and respiratory health	TA	2 wks acclim. 2x 8-hour trips, 48hrs apart	12	3-8yrs	M,G	Balance, stress & other behav.	E3c	PS, PHYS	PC
Siniscalchi *et al.* 2014	Forelimb preference when loading	TA	10 weeks – trials once a week	14	Average 6yrs	M,G	Stress behaviour. L/R forelimb on ramp	E2c	PS	HR
Stewart *et al.* 2003	Transport by air	TA	Short (3-4hr) & long haul (10-15hr) flights	16	2-9yrs	M,G, S	Social, stance, balance, eating	E3c	PS	HR,RT
Tateo *et al.* 2012	Journey distance	RM	Short (50km) & long (200km) road journey & 4hrs post t	12	3-7yrs	M,G, S	Balance movements in transport Behavioural states / events post t	E2a	PS PHYS	HR,PC,RR,RT
Waran & Cuddeford 1995	Loading, transport effects	BS,RM 2-part study	1 x loading, 2x 25mins in lorry, with 7 days in between trials	32, 7	1-20yrs	Not given	Evasions when loading, position and events in transit	E3a	PS, PHYS	HR

Table S2.6 References:

Hendriksen P, Elmgreen K and Ladewig J 2011 Trailer-loading of horses: Is there a difference between positive and negative reinforcement concerning effectiveness and stress-related signs? *Journal of Veterinary Behavior: Clinical Applications and Research* **6(5):** 261–266. https://doi.org/10.1016/j.jveb.2011.02.007

Ji SN, Yang LL, Ge XF, Wang BJ, Cao J and Hu DF 2013 Behavioural and physiological stress responses to transportation in a group of Przewalski's horses (*Equus ferus prezwalskii*). *Journal of Animal and Plant Sciences* **23**: 1077–1084

Kay R and Hall C 2009 The use of a mirror reduces isolation stress in horses being transported by trailer. *Applied Animal Behaviour Science* **116**: 237–243. https://doi.org/10.1016/j.applanim.2008.08.013

Knowles TG, Brown SN, Pope SJ, Nicol CJ, Warriss PD and Weeks CA 2010 The response of untamed (unbroken) ponies to conditions of road transport. *Animal Welfare* **19**: 1–15. https://doi.org/10.1017/S096272860000110X

Munsters CCBM, De Gooijer JW, Van Den Broek J and Van Oldruitenborgh-Oosterbaan MMS 2013 Heart rate, heart rate variability and behaviour of horses during air transport. *Veterinary Record* **172**: 15-15. https://doi.org/10.1136/vr.100952

Padalino B, Maggiolino A, Boccaccio M and Tateo A 2012 Effects of different positions during transport on physiological and behavioral changes of horses. *Journal of Veterinary Behavior: Clinical Applications and Research* **7**: 135–141. https://doi.org/10.1016/j.jveb.2011.09.003

Padalino B and Raidal SL 2020 Effects of transport conditions on behavioural and physiological responses of horses. *Animals* **10**: 1–18. https://doi.org/10.3390/ani10010160

Padalino B, Raidal SL, Knight P, Celi P, Jeffcott L and Muscatello G 2018 Behaviour during transportation predicts stress response and lower airway contamination in horses. *PLoS ONE* **13**: 1–20. https://doi.org/10.1371/journal.pone.0194272

Siniscalchi M, Padalino B, Lusito R and Quaranta A 2014 Is the left forelimb preference indicative of a stressful situation in horses? *Behavioural Processes* **107**: 61–67. https://doi.org/10.1016/j.beproc.2014.07.018

Stewart M, Foster TM and Waas JR 2003 The effects of air transport on the behaviour and heart rate of horses. *Applied Animal Behaviour Science* **80:** 143–160. https://doi.org/10.1016/S0168-1591(02)00212-5

Tateo A, Padalino B, Boccaccio M, Maggiolino A and Centoducati P 2012 Transport stress in horses: Effects of two different distances. *Journal of Veterinary Behavior: Clinical Applications and Research* **7(1):** 33–42. https://doi.org/10.1016/j.jveb.2011.04.007

Waran NK and Cuddeford D 1995 Effects of loading and transport on the heart rate and behaviour of horses. *Applied Animal Behaviour Science* **43(2):** 71–81. https://doi.org/10.1016/0168-1591(95)00555-7

Table S2.7. Study details for the retained articles (n = 9) relating to the behaviour of the horse during training other than when ridden (see Section 1 above for the key to abbreviations used)

Study	Focus of study and other tests included	Study design	Length of study	Subject details			Behaviour recorded	Type of ethogram used	Supporting evidence	Physiological measures
				No.	Age	Sex				
Eisersiö *et al.* 2021	Rein tension & training	RM	3 days total, for each horse both treatments consec.	20	4-5yrs, Average 10.3yrs	M,G	Backing response, head/neck movement, attention	E3c	PS, rein tension measured	
Eisersiö *et al.* 2023	Rein tension	RM	8 trials per horse consec.	20	Average 7.5yrs	Not spec.	Oral behaviour, head movement	E3a	PS, rein tension measured	
Fenner *et al.* 2016	Tight nosebands	RM	Each horse tested over 4 consec. days – total 3 weeks (10 mins)	12	Average 6.6yrs	M,G, S	Head, mouth movements. Re-bound (licking, swallowing, yawning)	E3a	PS, PHYS	HR,HRV,ET
Fureix *et al.* 2009	Training type (traditional vs. natural horsemanship)	BS	4 sessions 50 mins, and pre- / post-tests	12	2-10yrs	M	Reactions to isolation, bridge test, neophobia, human approaches	E3a	PS, APP	
Larssen & Roth 2022	+ve reinforcement, human approach	BS	9 week +ve reinforceme	36	Average 10-11yrs	M,G	Human contact	E3a	PS, APP	HC

	& cognitive bias test		nt training then tests				seeking, cog. bias			
Nittynen *et al.* 2022	Foundation training	TA	9 months training	19	1,2,3yrs	G,F,C	Reactivity, fear, contact	E3c]	PS, APP, PHYS	SOX,SC
Rietmann *et al.* 2004b	Walking backwards	RM	3 days	20	5-14yrs	M,G	Head position, movement, tail swishing, defaecation	E3c	PS, PHYS	HR,HRV
Sankey *et al.* 2010	+ve/-ve reinforcement, human approach test	BS	5 days training, 5 months to follow-up	21	10-16yrs	M,G	Human contact seeking	E3c	PS, PHYS	HR
Smiet *et al.* 2014	Head-neck position	RM	5 x tests (34 mins each) 1 per day	7	Average 10yrs	M,G	Conflict behaviours	E3c	PS, PHYS	HR,HRV,SC, PC

Table S2.7 References

Eisersiö M, Yngvesson J, Byström A, Baragli P and Egenvall A 2021 A rein tension signal can be reduced by half in a single training session. *Applied Animal Behaviour Science* **243:** May. https://doi.org/10.1016/j.applanim.2021.105452

Eisersiö M, Yngvesson J, Hartmann E and Egenvall A 2023 Gaping for relief? Rein tension at onset and end of oral behaviors and head movements in unridden horses. *Journal of Veterinary Behavior* **59**: 8–14. https://doi.org/10.1016/j.jveb.2022.11.009

Fenner K, Yoon S, White P, Starling M and McGreevy P 2016 The effect of noseband tightening on horses' behavior, eye temperature, and cardiac responses*. PLoS ONE* **11:** 1–20. https://doi.org/10.1371/journal.pone.0154179

Fureix C, Pagès M, Bon R, Lassalle JM, Kuntz P and Gonzalez G 2009 A preliminary study of the effects of handling type on horses' emotional reactivity and the human-horse relationship. *Behavioural Processes* **82:** 202–210. https://doi.org/10.1016/j.beproc.2009.06.012

Larssen R and Roth LSV 2022 Regular positive reinforcement training increases contact-seeking behaviour in horses. *Applied Animal Behaviour Science* **252**: 105651. https://doi.org/10.1016/j.applanim.2022.105651

Niittynen T, Riihonen V, Moscovice LR and Koski SE 2022 Acute changes in oxytocin predict behavioral responses to foundation training in horses. *Applied Animal Behaviour Science* **254**: 105707. https://doi.org/10.1016/j.applanim.2022.105707

Rietmann TR, Stuart AEA, Bernasconi P, Stauffacher M, Auer JA and Weishaupt MA 2004b. Assessment of mental stress in warmblood horses: Heart rate variability in comparison to heart rate and selected behavioural parameters. *Applied Animal Behaviour Science* **88**: 121–136. https://doi.org/10.1016/j.applanim.2004.02.016

Sankey C, Richard-Yris MA, Henry S, Fureix C, Nassur F and Hausberger M 2010 Reinforcement as a mediator of the perception of humans by horses (*Equus caballus*). *Animal Cognition* **13**: 753–764. https://doi.org/10.1007/s10071-010-0326-9

Smiet E, Van Dierendonck MC, Sleutjens J, Menheere PPCA, van Breda E, de Boer D, Back W, Wijnberg ID and Van Der Kolk JH 2014 Effect of different head and neck positions on behaviour, heart rate variability and cortisol levels in lunged Royal Dutch Sport horses. *The Veterinary Journal* **202**: 26–32. https://doi.org/10.1016/j.tvjl.2014.07.005
